# Using genetic markers to orient the edges in quantitative trait networks: The NEO software

**DOI:** 10.1186/1752-0509-2-34

**Published:** 2008-04-15

**Authors:** Jason E Aten, Tova F Fuller, Aldons J Lusis, Steve Horvath

**Affiliations:** 1 Human Genetics, David Geffen School of Medicine, University of California, Los Angeles, USA; 2 Biomathematics, David Geffen School of Medicine, University of California, Los Angeles, USA; 3 Microbiology, Immunology and Molecular Genetics, University of California, Los Angeles, USA; 4 Biostatistics, School of Public Health, University of California, Los Angeles, USA

## Abstract

**Background:**

Systems genetic studies have been used to identify genetic loci that affect transcript abundances and clinical traits such as body weight. The pairwise correlations between gene expression traits and/or clinical traits can be used to define undirected trait networks. Several authors have argued that genetic markers (e.g expression quantitative trait loci, eQTLs) can serve as causal anchors for orienting the edges of a trait network. The availability of hundreds of thousands of genetic markers poses new challenges: how to relate (anchor) traits to multiple genetic markers, how to score the genetic evidence in favor of an edge orientation, and how to weigh the information from multiple markers.

**Results:**

We develop and implement Network Edge Orienting (NEO) methods and software that address the challenges of inferring unconfounded and directed gene networks from microarray-derived gene expression data by integrating mRNA levels with genetic marker data and Structural Equation Model (SEM) comparisons. The NEO software implements several manual and automatic methods for incorporating genetic information to anchor traits. The networks are oriented by considering each edge separately, thus reducing error propagation. To summarize the genetic evidence in favor of a given edge orientation, we propose Local SEM-based Edge Orienting (LEO) scores that compare the fit of several competing causal graphs. SEM fitting indices allow the user to assess local and overall model fit. The NEO software allows the user to carry out a robustness analysis with regard to genetic marker selection. We demonstrate the utility of NEO by recovering known causal relationships in the sterol homeostasis pathway using liver gene expression data from an F2 mouse cross. Further, we use NEO to study the relationship between a disease gene and a biologically important gene co-expression module in liver tissue.

**Conclusion:**

The NEO software can be used to orient the edges of gene co-expression networks or quantitative trait networks if the edges can be anchored to genetic marker data. R software tutorials, data, and supplementary material can be downloaded from: .

## Background

The pairwise relationships between different clinical traits (e.g. cholesterol level) and/or gene expression traits (e.g. mRNA levels) have been successfully described with undirected gene co-expression networks [1-11]. While gene expression traits (profiles) and clinical traits represent different quantities, both can be described in undirected *trait networks*. By definition, these undirected networks cannot be used to describe causal relationships between the traits. Causal information can be encoded by directed networks where *A *→ *B *if trait *A *causally influences trait *B*. We refer to the process of assigning a causal direction to at least some of the edges in a trait network as 'edge orienting'. Experimental edge orienting approaches include transgenic modifications, viral-mediated over-expression, and chemical perturbation of genes. Edge orienting methods can also be based on various approaches that involve multiple perturbations, such as genetic- and time series experiments [12], or by integrating protein interaction and gene expression data [13].

Using genetic markers for orienting the edges of trait networks generated in genetic experiments provides significant statistical power and specificity for recovering directed edges [14-22]. Since randomization is the most convincing method for establishing causal relationships between two traits [23,24], it is natural to make use of genetically randomized genotypes (implied by Mendel's laws) to derive causality tests that are less susceptible to confounding by hidden variables [19,25-29]. If a trait *A *is significantly associated with a genetic marker *M*, variation in *M *must be a cause of variation in *A *(denoted by *M *→ *A*) since the randomization of marker alleles during meiosis precedes their effect on trait *A*. Since the orientation of the edge between *M *and *A *is unambiguous, *M *is referred to as a causal anchor of *A *[15].

We follow the convention of path analysis to represent a causal model by a directed graph. For example, the directed graph *M *→ *A *→ *B *implies that the genetic marker *M *has a causal effect on trait *A*, which in turn has a causal effect on trait *B*. A causal graph encodes independencies between variables. Conditional independence can be determined by the graphical property of d-separation [30-32]. If two traits *A *and *B *are d-separated in the graph by a set of variables *S*, then the two traits are independent given the variables in *S*. For example, *M *→ *A *→ *B *implies that *M *and *B *are independent after conditioning on *A*.

D-separation predicts the correlational consequences of conditioning in the causal graph [30]. By testing the correlational predictions and assuming no false independencies (faithfulness assumption), one can sometimes orient edges using observational data alone [31-39].

## Results

### Correlation-based tests of causal models

For simplicity, we assume that the genetic markers are single nucleotide polymorphisms (SNPs). For a given sample (e.g. a mouse), a bi-allelic SNP can take on one of three possible genotypes. By default, we assume an additive genetic effect and encode these genotypes as 0, 1, or 2, but alternative marker codings could also be considered. To quantify the linear relationship between a SNP marker *M *and a trait *A*, we use the correlation coefficient *cor*(*M*, *A*). Ordinal variables are routinely used in path analysis and structural equation modelling [32,40].

To determine whether trait *A *mediates the effect of marker *M *on trait *B *(*M *→ *A *→ *B*) one can assess how conditioning on *A *affects the correlation between *M *and *B*. To quantify the linear relationship between *M *and *B *after conditioning on *A*, we use the partial correlation coefficient:

(1) $$cor(M,B|A)=\frac{cor(M,B)-cor(M,A)cor(B,A)}{\sqrt{\left(1-cor{\left(M,A\right)}^{2})(1-cor{\left(B,A\right)}^{2}\right)}}.$$

If the causal model *M *→ *A *→ *B *is correct, then the partial correlation coefficient *cor*(*M*, *B*|*A*) is expected to be 0.

We use Fisher's Z transform to assess the statistical significance of a sample correlation coefficient *r *[23]:

$$Z_{Fisher}(r)=0.5\sqrt{N-3}\;log\left(\frac{1+r}{1-r}\right),$$

where *N *denotes the sample size; *Z*_*Fisher*_(*r*) asymptotically follows a normal distribution (*Normal*(*μ*, 1) with mean *μ *and variance 1. Under the null hypothesis of zero correlation, *μ *= 0 and *Z*_*Fisher*_(*r*) follows a standard normal distribution. For brevity, we denote the Fisher transformations of the correlation coefficients *cor*(*A*, *B*) and *cor*(*M*, *B*|*A*) by *Z*(*A*, *B*) = *Z*_*Fisher*_(*cor*(*A*, *B*)) and *Z*(*M*, *B*|*A*) = *Z*_*Fisher*_(*cor*(*M*, *B*|*A*)), respectively.

**Figure 1:**
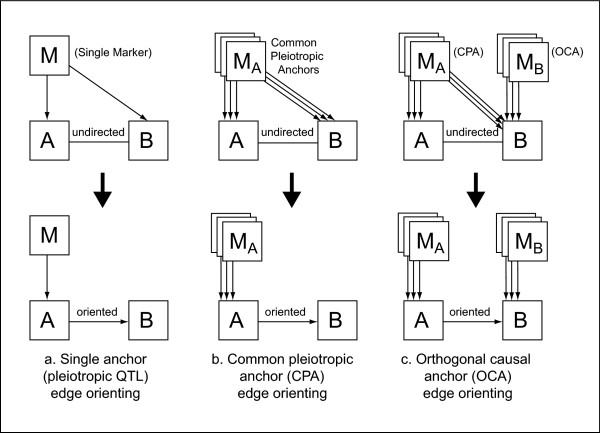
**Approaches for genetic marker-based causal inference**. Here we contrast different approaches for causality testing based on genetic markers. (a) single marker edge orienting involving a candidate pleiotropic anchor (CPA) *M*. The upper half of (a) shows the starting point of network edge orienting based on a single genetic marker *M *which is associated with traits *A *and *B*. The undirected edge between *A *and *B *indicates a significant correlation *cor*(*A*, *B*) between the two traits. The causal model in the lower half of (a) implies the following relationship between the correlation coefficients *cor*(*M*, *B*) = *cor*(*M*, *A*) × *cor*(*A*, *B*). Further it implies that the absolute value of the correlations |*cor*(*M*, *A*)| and |*cor*(*M*, *B*)| are high whereas the partial correlation |*cor*(*M*, *B*|*A*)| (Eq. 1) is low. Figure (b) generalizes the single marker situation to the case of multiple genetic markers $M_{A}=\{M_{A}^{\left(1\right)},M_{A}^{\left(2\right)},...\}$. In this case, it is straightforward to generalize single edge orienting scores to multi-marker scores. Figure (c) describes a situation when a set of genetic markers $M_{B}=\{M_{B}^{\left(1\right)},M_{B}^{\left(2\right)},...\}$ is also available for trait *B*. We refer to the *M*_*B *_markers as orthogonal causal anchors (OCA) since $cor(A,M_{B}^{\left(j\right)})$ is expected to be 0 under the causal model *M*_*A *_→ *A *→ *B *→ *M*_*B*_, the correlation. Using simulation studies, we find that edge scores based on OCAs can be more powerful than those based on CPAs (see Additional File 1).

If the causal graph *M *→ *A *→ *B *(Figure 1a) is correct, *Z*(*M*, *B*|*A*) follows a standard normal distribution. Thus, if the p-value corresponding to *Z*(*M*, *B*|*A*) is high (non-significant), the data fit the assumed causal graph. Using path analysis rules, the causal graph *M *→ *A *→ *B *(Figure 1a) implies the following relationships between the correlation coefficients

(2) *cor*(*M*, *B*) = *cor*(*M*, *A*)*cor*(*A, B*)

We refer to the marker *M *as a *candidate common pleiotropic anchor *(CPA) of *A *and *B*. If the expected values of *cor*(*M*, *A*) and *cor*(*A*, *B*) are non-zero and the causal model holds, Eq. (2) implies that the genetic marker *M *will be significantly correlated with both *A *and *B*. Thus, the marker *M *can be confirmed as a pleiotropic anchor of *A *and *B *by confirming the fit of the causal model *M *→ *A *→ *B*. We will now consider a situation where the correlation between *A *and *B *stems from a hidden confounder *C*, i.e. *M *→ *A *← *C *→ *B*. The graph implies that *A *and *B *are correlated due to the shared confounder *C*. The correlation *cor*(*M*, *B*) is expected to be 0 since the arrows between *M *and *B *collide at *A*, i.e, *M *and *B *are d-separated without conditioning. In this situation *Z(M, B) = Z_Fisher_(cor(M, B))* follows a standard normal distribution. If the p-value corresponding to Z(M, B) is high (non-significant), the data fit a confounded model. In contrast, the partial correlation *cor*(*M*, *B*|*A*) is expected to be non-zero since conditioning on *A *'activates' the causal flow through the collider node, i.e. it induces conditional dependence [32].

The opposite (reactive) causal graph *M *→ *A *← *B *also implies that the expected value of *cor*(*M*, *B*) is zero since the causal paths collide at *A*. Conditioning on *A *activates this collider node, and the partial correlation *cor*(*M*, *B*|*A*) is expected to be non-zero.

Similarly, one can show that the model *A *← *M *→ *B *implies that *cor*(*A*, *B*|*M*) is expected to be zero. Under this causal model, *Z*(*A*, *B*|*M*) asymptotically follows a standard normal distribution. In contrast, *cor*(*M*, *B*) is expected to be non-zero.

These considerations illustrate that one can test the predicted correlational consequences of a causal model and thus evaluate its fit.

We will now consider the situation of multiple markers (Figure 1b). Denote by

(3) $$M_{A}=\{M_{A}^{\left(1\right)},M_{A}^{\left(2\right)},...M_{A}^{\left(K_{A}\right)}\}$$

a set of candidate common pleiotropic anchors of *A *and *B*. Analogous to Eq. (2), the causal model *M*_*A *_→ *A *→ *B *implies $cor(M_{A}^{\left(i\right)},B)=cor(M_{A}^{\left(i\right)},A)cor(A,B)$. The model implies that the partial correlations $cor(M_{A}^{\left(i\right)},B|A)$ are expected to be zero, i.e. $Z_{fisher}(cor(M_{A}^{\left(i\right)},B|A))$ is predicted to follow a standard normal distributions.

Frequently an additional set of markers *M*_*B *_is also available for trait *B *(Figure 1c). For example, when one marker is available for each trait, i.e. $M_{A}^{\left(1\right)}\to A\to B\leftarrow M_{B}^{\left(1\right)}$, the correlation $cor(A,M_{B}^{\left(1\right)})$ is expected to be 0 since the causal arrows 'collide' at *B *[30]. Geometrically speaking, the expected zero correlation between *A *and $M_{B}^{\left(1\right)}$ implies that the corresponding standardized vectors are orthogonal. Therefore, we refer to marker $M_{B}^{\left(1\right)}$ as an **orthogonal causal anchor **(OCA) with respect to the edge *A *→ *B*. We will argue that the availability of orthogonal causal anchors significantly improves the recovery of the causal signal (see the simulations in Additional File 1). If the model $M_{A}^{\left(1\right)}\to A\to B\leftarrow M_{B}^{\left(1\right)}$ is correct, $cor(M_{A}^{\left(1\right)},B|A),cor(A,M_{B}^{\left(1\right)})$ are expected to be zero and $Z(M_{A}^{\left(1\right)},B|A)$ and $Z(A,M_{B}^{\left(1\right)})$ asymptotically follow standard normal distributions.

Figure 1(c) depicts a situation where two sets of genetic markers $M_{A}=\{M_{A}^{\left(1\right)},M_{A}^{\left(2\right)},...M_{A}^{\left(K_{A}\right)}\}$ and $M_{B}=\{M_{B}^{\left(1\right)},M_{B}^{\left(2\right)},...M_{B}^{\left(K_{B}\right)}\}$ influence traits *A *and *B*, respectively. In this case, the correlational consequences become increasingly complicated, which is why we use structural equation models (SEMs) to evaluate the fit of different causal scenarios.

### Local SEM-based edge orienting scores

**Figure 2:**
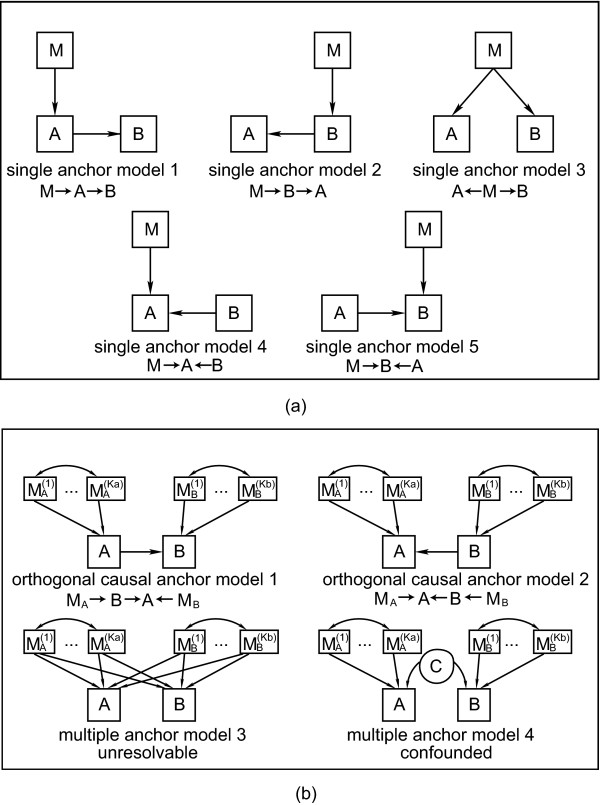
**Illustrating the single genetic marker versus multi-marker local SEMs used in the definition of the LEO.NB score**. The single genetic marker is denoted by *M *in (a) and the multiple genetic markers are denoted by $M_{A}^{\left(i\right)}$ and $M_{B}^{\left(j\right)}$ in (b) and (c). By definition, *LEO.NB*(*A*˃*B*) = log_10_{*P *(model 1)}/{max_*i*>1_{*P *(model *i*)}} for a candidate *A *→ *B *edge orientation, where the models in the definition are pictured in (a) for single marker LEO.NB scores, and in (b) for multiple marker LEO.NB scores. In (b) we show the orthomarker models used for the LEO.NB.OCA marker aggregation method. The hidden confounder *C *in model 4 is the causal parent of both *A *and *B*, i.e. *A *← *C *→ *B*. The simulation studies in Additional File 1 show that the LEO.NB.OCA score can be significantly more powerful than the LEO.NB.CPA score.

While SEMs can be used to study the fit of multi-trait causal models [17,20] we only consider the *local causal models *depicted in Figure 2 since the proposed NEO method evaluates the orientation of each edge separately based on the best causal anchors available. The fit of each single marker model in Figure 2(a) can be tested using a chi-square test with 1 degree of freedom. We refer to the resulting p-value as the model p-value. In the Methods section, we review and discuss the use of model p-values for quantifying the fit of a causal model. The main point is that the *higher *the model p-value, the better the causal model fits the data.

To summarize the genetic evidence in favor of a given edge orientation *A *→ *B*, we propose the use of edge orienting scores. The higher the value of an edge orienting score for the orientation *A *→ *B*, the stronger genetic evidence favors this causal model.

In the following, we propose local SEM-based edge orienting (LEO) scores for orientation *A *→ *B*. For a single genetic marker *M *and traits *A *and *B*, we consider the 5 different local causal models depicted in Figure 2(a). Additional single marker models are possible. However, under the constraint that the markers are causal anchors (graphically, arrows flow only from M and not into M), then the five models pictured for nodes (M, A, B) in Figure 2(a) exhaust all possible three node models that both (1) explain *A *– *B *and (*M *– *A *or *M *– *B*) associations and (2) can be tested. The critical technical issue is having degrees of freedom (d.f.) remaining after estimating the model parameters. If the degrees of freedom are 0, the model p-values cannot be calculated. The 5 different local causal models depicted in Figure 2(a) are used to compute the following model p-values: *P*(*M *→ *A *→ *B*), *P*(*A *← *B *← *M*), *P*(*A *← *M *→ *B*), *P*(*M *→ *A *← *B*), and *P*(*A *→ *B *← *M*). While a detailed analysis should consider all model p-values, we find it useful to summarize the genetic evidence in favor of a given orientation *A *→ *B *(model 1) using a single number: the Local SEM-based Edge Orienting Next Best (LEO.NB) score. The LEO.NB score is defined by dividing the model p-value for *A *→ *B *by the p-value of the best fitting alternative model, i.e. the best of models 2–5 in Figure 2(a). The chi-square test p-value of the best fitting alternative model is the maximum p-value of the alternative causal models. Specifically, we define the single-marker LEO.NB score as follows:

(4) $$LEO.NB.SingleMarker(A\to B|M)=\log_{10}\left(\frac{P(\text{model }1:M\to A\to B)}{\max\left(\begin{array}{l} P(\text{model }2:M\to B\to A),\\ P(\text{model }3:A\leftarrow M\to B),\\ P(\text{model }4:M\to A\leftarrow B),\\ P(\text{model }5:M\to \text{B}\leftarrow \text{A)} \end{array}\right)}\right).$$

A positive *LEO.NB*(*A *→ *B*) score indicates that the p-value in favor of model *A *→ *B *is higher than that of any of the competing models in Figure 2(a). A negative LEO.NB score indicates that the *A *→ *B *model is inferior to at least one alternative model. In our simulations, we use a threshold of 1 for *LEO.NB.SingleMarker*(*A *→ *B*|*M*).

#### Multi-marker LEO.NB score

It is straightforward to generalize the single marker LEO.NB score (Eq. 4) to a set of genetic markers $M_{A}=\{M_{A}^{\left(1\right)},M_{A}^{\left(2\right)},...\}$ (Figure 1b). We refer to the resulting edge orienting score as the LEO.NB.CPA score since it is based on the set of candidate pleiotropic anchors *M*_*A *_(Eq. 3):

(5) $$LEO.NB.CPA(A\to B|M_{A})=\log_{10}\left(\frac{P(\text{model }1:M_{A}\to A\to B)}{\max\left(\begin{array}{l} P(\text{model }2:M_{A}\to B\to A),\\ P(\text{model }3:A\leftarrow M_{A}\to B),\\ P(\text{model }4:M_{A}\to A\leftarrow B),\\ P(\text{model }5:M_{A}\to \text{B}\leftarrow \text{A)} \end{array}\right)}\right).$$

Note that the multi-marker models used in the definition of LEO.NB.CPA correspond to the single marker models of Figure 2(b) with *M *replaced by *M*_*A*_.

If an additional genetic marker set $M_{B}=\{M_{B}^{\left(1\right)},M_{B}^{\left(2\right)},...M_{B}^{\left(K_{B}\right)}\}$ associated with trait *B *is available (Figure 1c), we propose to use another edge orienting score. According to our consistency assumption, *M*_*A *_contains markers that are more strongly correlated with *A *than with *B*. Similarly, *M*_*B *_holds markers more strongly correlated with *B *than with *A*. If the orientation *A *→ *B *is correct, then each of the *M*_*A *_markers has a pleiotropic effect by impacting first *A *and subsequently *B*. Furthermore, we refer to the markers in *M*_*B *_as candidate orthogonal causal anchors (OCAs) since the model *A *→ *B *implies that these markers impact *B*, but are independent of both *A *and *M*_*A*_. We define the likelihood-based orthogonal causal anchor (OCA) score by assessing whether the model *M*_*A *_→ *A *→ *B *← *M*_*B *_has a higher p-value than the alternative models depicted in Figure 2(b). Specifically, we define

(6) $$LEO.NB.OCA(A\to B|M_{A},M_{B})=\log_{10}\left(\frac{P(\text{model }1:M_{A}\to A\to B\leftarrow M_{B})}{\max\left(\begin{array}{l} P(\text{model }2:M_{A}\to A\leftarrow B\leftarrow M_{B}),\\ P(\text{model }3:B\leftarrow M_{A}\to A;A\leftarrow M_{B}\to B),\\ P(\text{model }4:M_{A}\to A\leftarrow C\to B\leftarrow M_{B}) \end{array}\right)}\right).$$

Note that model 4 in the denominator involves a hidden confounder *C*. The use of two independent genetic marker sets (*M*_*A *_and *M*_*B*_) alleviates the problem of model identifiability that may plague a CPA based edge orienting score.

Model equivalence is also a key consideration in choosing which models to compare. From the standpoint of model equivalence, we note that the multiple anchor models presented in Figure 2(b) include a model with a hidden (latent) variable connecting *A *and *B*, and that no such model is included in the single anchor model comparisons. Such a model was found to be indistinguishable from the models with a collider node, such as single anchor models 4 and model 5. In the single marker case, both the collider node and the hidden variable models test for independence in the marginal relationship between the anchor and the more distal trait node. Future research may lead to an understanding of what type of data allow one to consider additional alternative models for the edge score computation. It should be straightforward to adapt the proposed LEO score to additional models as long as their model p-values can be calculated. Correlated markers, which are frequently encountered in practice such as in haplotype blocks, may compromise the performance of edge orienting scores. LEO scoring allows multiple parents of a node to be correctly accounted for within each model. Moreover, the parents (causal anchors) of a model are allowed to co-vary. By contrast, the orthogonal causal anchor set is, by definition, penalized for any covariation with the pleiotropic anchors.

#### Thresholds for the edge orienting scores

For the single marker score *LEO.NB.SingleMarker*, we use a threshold of 1, which implies that the model p-value of the causal model is 10^1 ^= 10 fold higher than that of the next best model. For the LEO.NB.CPA and the LEO.NB.OCA, we use lower thresholds of 0.8 and 0.3, respectively. Using simulation studies presented in the Additional File, we found that these thresholds lead to false positive rates that are often substantially below 0.05. Similar to other statistical procedures, NEO is susceptible to the pitfalls of multiple testing that may inflate the false positive rate. Permutation procedures and data dependent schemes (e.g. based on the false discovery rate) may inform the user on how to pick a threshold for a particular application. Further, we provide R software code for carrying out both single edge and multi-edge simulation studies. Simulation studies can be used to determine the power and false positive rates in different settings (sample size, causal signal, confounders, etc).

In practice, one often observes strong dependence relationships between genetic markers. Our simulations show that correlations between genetic markers can reduce the power of edge orienting scores. Further, we mention that the NEO software implements an option for removing redundant markers that are highly correlated with each other. The removal of redundant markers may alleviate the loss of power.

### Overview of network edge orienting with NEO

**Figure 3:**
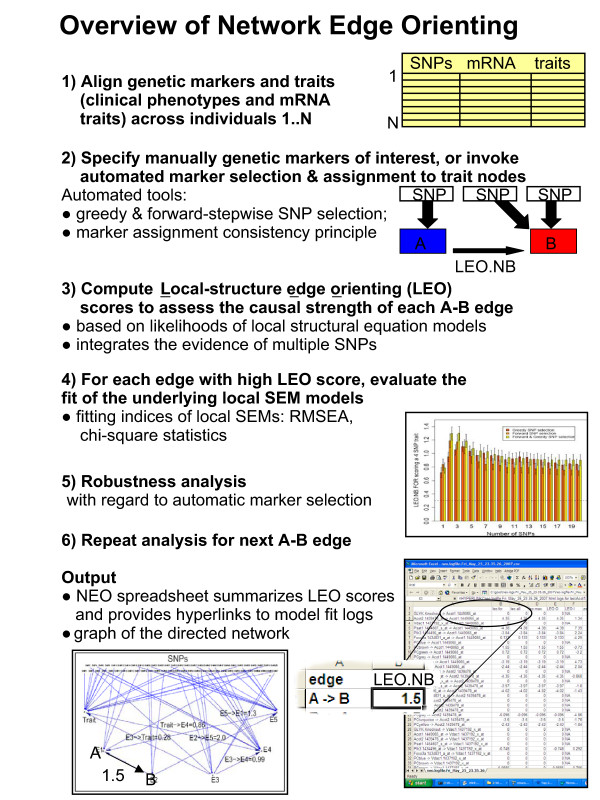
**Overview of the network edge orienting method**. The steps of the network overview analysis are described in the text.

We now provide a detailed step-by-step description of a typical NEO analysis. An overview is also provided in Figure 3.

#### Step 1: Integrate traits (gene expression traits and clinical traits) and SNPs

NEO takes trait and genetic marker data as input. Traits can include microarray gene expression data, clinical phenotypes, or other quantitative variables. Each SNP or trait is a node in the network, and the NEO software evaluates and scores the edge between traits *A *and *B *if the absolute correlation |*cor*(*A*, *B*)| lies above a user-specified threshold. For each edge *A *– *B*, NEO generates edge orienting scores for both possible orientations: *A *→ *B *and *B *→ *A*. If an erroneous edge exists between two traits, then it is meaningless to orient it. The NEO software can be used to orient any edge that the user chooses to consider. To allow the user to judge whether the existence of an edge is supported by the data, the NEO software outputs a Wald test statistic of the path coefficient, the corresponding p-value, and the correlation between the two traits. If the Wald test p-value is insignificant, orienting the edge may be meaningless.

#### Step 2: Genetic marker selection and assignment to traits

Edge orienting scores will only be generated for edges whose traits have been anchored to at least one genetic marker. Two basic approaches for anchoring traits to markers are implemented in the NEO software: a manual selection by the user or an automatic selection by the software itself.

##### Manual SNP selection

**Figure 4:**
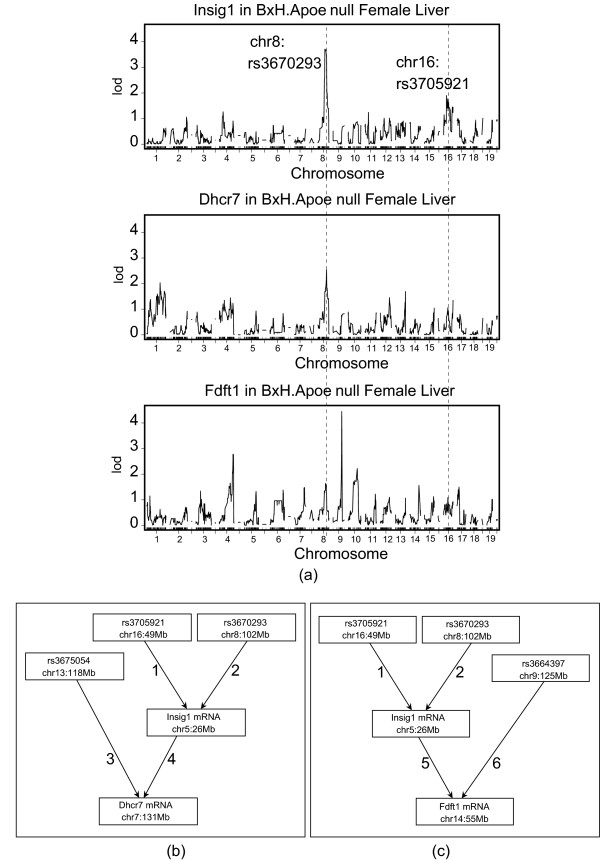
**Manual SNP selection to study Insig1 → Dhrc7 and Insig1 → Fdft1 in mouse liver**. Using female liver gene expression data and SNP markers from the BxH mouse intercross, NEO retrieves known causal relationships in the cholesterol biosynthesis pathway: *Insig1 → Dhrc7 *and *Insig1 → Fdft1*. The single marker LOD score curves in (a) motivate our choice of manually selected SNPs (one SNP on chromosome 16 and another on chromosome 8). These SNP markers can also be used to screen for genes that are reactive to *Insig1*, see Table 2. Figures (b) and (c) show the causal models used to compute the model p-values in favor of edge orientations *Insig*1 → *Dhcr*7 and *Insig*1 → *Fdft*1, respectively. More details on the individual edges are presented in Table 1.

NEO provides great flexibility to the user on how to anchor traits to markers. For example, the user can manually assign SNPs to the traits (see the example in Figure 4). This flexibility entails that the user carefully studies what constitutes a significant relationship between traits and markers and between the traits in the data set. The user may wish to anchor traits to SNPs that have been implicated by prior genetic analyses. For example, results from previous quantitative trait locus studies may implicate genetic markers associated with a trait. Multiple comparison issues are just starting to be addressed in the SEM literature [39,41,42]. Edge scores cannot be computed when an overly strict multiple testing control results in no causal anchors. On the other hand, an overly lax multiple testing control may result in spurious causal anchors which may lead to erroneous edge scores. We recommend that conservative measures of genome-wide QTL significance [43] and false discovery rate be applied when selecting the initial causal anchor(s). Once a causal anchor has been established as obtaining genome-wide significance, NEO can be used to evaluate the fit of different causal models.

##### Automatic SNP selection

NEO can also be used to automatically relate (anchor) traits to SNPs. The automated SNP selection methods consider each trait *A *in isolation from the other traits when defining a *preliminary genetic marker set *(denoted by *M'*_*A*_). Toward this end, the user can choose 1) a greedy approach based on univariate linear regression models, 2) a forward-stepwise approach based on multivariate linear regression models, or 3) both. The greedy SNP selection approach defines *M'*_*A *_as the set of markers with the *K *highest absolute correlations with *A*. The greedy approach is equivalent to using univariate linear regression models to relate *A *to each marker separately and subsequently picking the *K *most significant markers.

For creating multivariate linear QTL models, NEO also implements forward-stepwise marker selection. The forward-stepwise marker selection method may avoid a pitfall that plagues the greedy SNP selection: if several genetic markers are located very close to each other (and are highly correlated), the greedy SNP selection may pick all of them before considering SNPs at other loci associated with the same trait. For this reason, we recommend combining greedy and forward-stepwise SNP selection methods.

Once the preliminary sets of markers *M*'_*A *_and *M'*_*B *_are obtained, NEO evaluates the consistency of each set. We utilize a *marker assignment consistency heuristic: *a genetic marker can only serve as causal anchor for one trait. To fulfill this heuristic, a SNP is moved from *M'*_*A *_to *M'*_*B *_if its correlation with *B *is stronger than that with trait *A*. We denote the resulting *consistent genetic marker sets *by *M"*_*A *_and *M"*_*B*_. The resulting consistent genetic marker sets may be comprised of dozens of SNPs. Therefore, it can be useful to further filter the SNPs according to their joint predictive power for the trait. Toward this end, we use the Akaike Information Criterion (AIC) in conjunction with multivariate regression models to select genetic markers from within the consistent genetic marker sets [44]. Specifically, to define the *final genetic marker set M*_*A *_for trait *A*, we use the AIC criterion to find a parsimonious multivariate regression model of *A *using predictors from within *M*"_*A*_. The final sets of markers *M*_*A *_and *M*_*B *_are thus comprised of consistent genetic markers that according to the AIC criterion best predict their respective traits; we use these final sets as causal anchors in computing the edge orienting scores.

The forward-stepwise approach based on multivariate linear regression models is akin to a legal courtroom where two cases are built, weighed, and judged. Broadly, the strongest genetic support (multivariate eQTL models) for the genetic influence on *A *and *B *are built independently, using AIC-based halting criteria. After consistency checks, these multivariate eQTL models are weighed by embedding them in causal models (one principal causal model in favor of edge orientation *A *→ *B *and alternative causal models) and models are then compared using SEM fitting indices. When candidate CPA markers can be found for *A *and OCAs for *B*, the NEO method provides stringent consistency checks and balances against over-fitting. We consider automated SNP selection particularly useful when no prior evidence suggests causal anchors for the traits.

#### Step 3: Compute local edge orienting scores for aggregating the genetic evidence in favor of a causal orientation

Both LEO.NB.CPA and LEO.NB.OCA scores are computed for each edge orientation (*A *→ *B *and *B *→ *A*). We recommend using the LEO.NB.OCA score (Eq. 6) as the primary edge orienting score if markers affect both *A *and *B*. However, if the results of the LEO.NB.CPA score strongly disagree with those of the LEO.NB.OCA score, the latter should not be trusted. As described in the next step, all fitting indices should be considered before calling an edge causal.

#### Step 4: For each edge, evaluate the fit of the underlying local SEM models

Edges with high edge orienting scores may not necessarily correspond to causal relationships. Although edge orienting scores flag interesting edges, they are no substitute for carefully evaluating the fit of the underlying local SEMs. Since a LEO.NB score is defined as a ratio of two model p-values, it is advisable to check whether both p-values are small, as this would indicate poor fit of either model. If the model p-value of the confounded model *A *← *C *→ *B *is high, the correlation between *A *and *B *may be largely due to a hidden confounder *C*. NEO (using the underlying *sem *R package) also report a Wald test statistic for the path coefficient from *A *→ *B*. If the Wald test for an edge is significant, the data support its existence. Apart from the model p-value, many other SEM model fitting indices have been defined by contrasting the observed covariance matrix *S*_*m *× *m *_with the fitted covariance matrix Σ($\hat{\theta }$) as detailed in the Methods section. The NEO software reports the standard SEM fitting indices [32,45] that are implemented in the R package *sem *[46] including the Root Mean Square Error of Approximation (RMSEA), Comparative Fit Index (CFI), Standardized Root Mean Square Residual (SRMSR), BIC. Since a single fitting index reflects only a particular aspect of model fit, a favorable value of that index does not by itself demonstrate good model fit; it is important to assess the model fit based on multiple indices. We follow the following standard guidelines for interpreting these indices [45]. Before calling an edge *A *→ *B *causal, we recommend verifying that the corresponding causal model has a high model p-value (say > 0.05), a low RMSEA score (say ≤ 0.05), a low SRMSR (say ≤ 0.10), a high CFI (say ≥ 0.90), and a significant Wald test p-value (say *p *≤ 0.05).

#### Step 5: Robustness analysis with respect to SNP selection parameters

**Figure 5:**
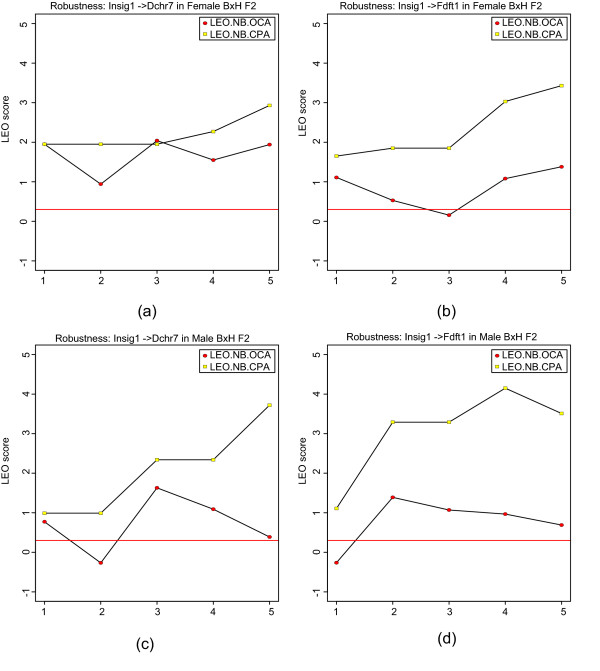
**Automatic SNP selection to score Insig1 → Dhrc7 and Insig1 → Fdft1 in female and male mouse livers**. These robustness plots show how the LEO.NB scores (y-axis) depend on sets of automatically selected SNP markers (x-axis). Here we use the default SNP selection method: combined greedy and forward stepwise method. Step *K *corresponds to choosing the top *K *greedy and top *K *forward selected SNPs for each trait. Since the greedy and the forward SNP selection may select the same SNPs, step *K *typically involves fewer than 2*K *SNPs per trait. Figures (a, b, top row) and (c, d) correspond to female and male BxH mice, respectively. Figures (a) and (c) report the results for edge *Insig*1 → *Dhrc*7 in female and male mouse livers, respectively. Figures (b) and (d) report the analogous results for *Insig*1 → *Fdft*1. NEO robustly retrieves the known causal relationship between these genes.

**Figure 6:**
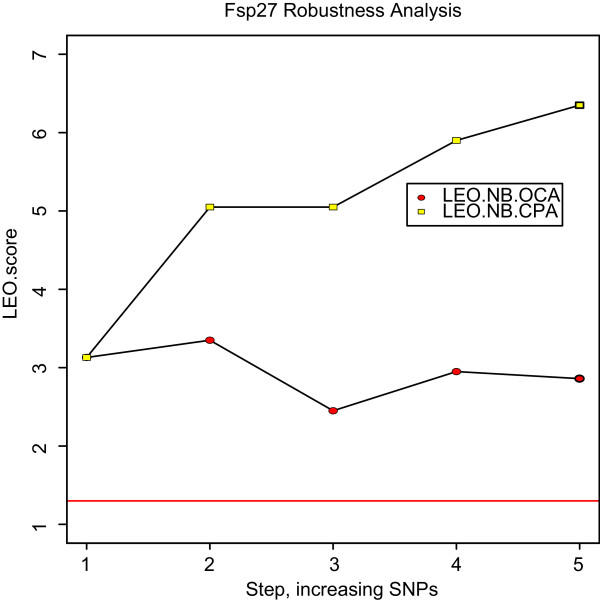
***Fsp27 *is a causal driver of a biologically important co-expression module**. Prior work using mouse liver expression data found the 'blue' co-expression module to be biologically important [7]. Here we used automatic SNP selection to determine whether *Fsp27 *is causal of the blue module gene expression profiles. The expression profiles of the blue module were summarized by their first principal component (referred to as module eigengene). The blue module eigengene *MEblue *can be considered as the most representative gene expression profile of the blue module. The figure shows the results of a robustness analysis regarding *LEO.NB*(*Fsp*27 → *MEblue*) (y-axis) with respect to different choices of genetic markers sets (x-axis). Both LEO.NB.CPA and LEO.NB.OCA scores show that the relationship is causal, i.e. the *Fsp*27 is upstream of the blue module expressions.

**Figure 7:**
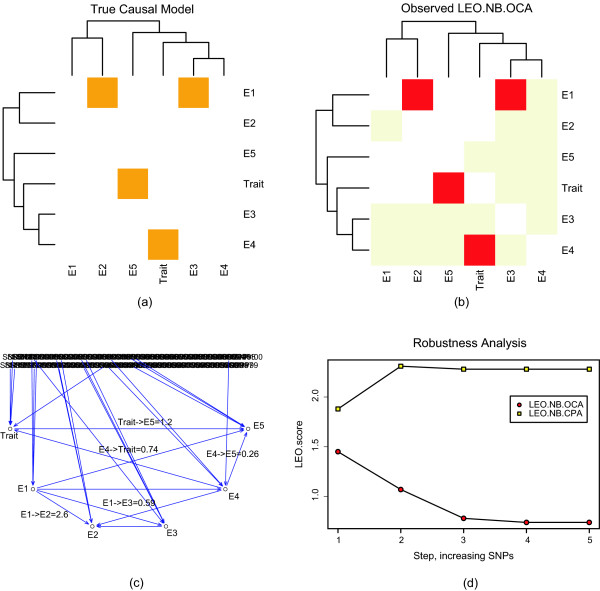
**Multi-edge simulation study involving 5 gene expression traits (*E*1-*E*5) and one clinical trait *Trait***. The heatmap plot in (a) depicts the true causal model. Note that a red square in the i-th row and j-th column indicates that trait *i *causally affects trait *j*, e.g. *E*1 → *E*2. The rows and columns of the heatmap are ordered according to a hierarchical clustering tree, which was constructed using average linkage hierarchical clustering based on the pairwise correlations of the traits. Figure (b) depicts the corresponding heatmap of the observed network that was reconstructed using the LEO.NB.OCA score. Figure (c) shows an alternative output graph of NEO. Blue edges indicate significant correlations and a LEO.NB.OCA score is added to each edges whose LEO.NB.OCA score passes a user-supplied threshold. We find that all true causal edges are correctly retrieved at the recommended LEO.NB.OCA threshold of 0.3. Figure (d) shows the results of a robustness analysis for the LEO.NB.OCA and LEO.NB.CPA scores for the edge orientation *E*4 → *Trait*. The LEO.NB.OCA scores exceed the recommended threshold of 0.3 (red horizontal line), i.e. they retrieve the orientation correctly. Similarly, the LEO.NB.CPA scores exceed the threshold of 0.8.

Since the edge orienting scores for an edge *A *– *B *critically depend on the input genetic marker sets *M*_*A *_and *M*_*B*_, we also recommend carrying out a robustness analysis with respect to different marker sets. In particular, the automated SNP selection results should be carefully evaluated with regard to the threshold parameters that were used to define the marker sets. For example, when using a greedy SNP selection strategy, it is advisable to study how the LEO.NB score is affected by altering the number of most highly correlated SNPs. For a given edge and a given edge orienting score (e.g. LEO.NB.OCA), NEO implements a robustness analysis with respect to automatic marker selection (see Figures 5, 6, and 7). A robustness plot shows how the LEO.NB.OCA score (y-axis) depends on sets of automatically selected SNP markers (x-axis). When using the default SNP selection method (combined greedy and forward stepwise method), robustness step *K *corresponds to choosing the top *K *SNPs by greedy and forward selection for each trait. Since the greedy and forward SNP selection may select the same SNPs, step *K *typically involves fewer than 2*K *SNPs per trait. The edge orienting results should be relatively robust with respect to different choices of *K*.

#### Step 6: Repeat the analysis for the next A-B trait-trait edge and apply edge score thresholds to orient the network

NEO orients each edge separately in an undirected input trait network. The results are order-independent. For each edge, NEO repeats steps 1–3 until all edges have been assigned edge orienting scores. Once each edge has been scored, the user can generate a global, directed network by choosing an edge score (e.g. LEO.NB.OCA) and a corresponding threshold (Figure 7).

#### NEO output and R software

The primary output of NEO is an Excel spreadsheet which reports likelihood-based edge scores (LEO.NB.CPA, LEO.NB.OCA) and other edge scores that are described in the NEO manual. For each edge, the NEO spreadsheet also contains hyperlinks that allows the user to access the log file for each edge. The log file contains a host of information regarding computation of the edge orienting scores including SEM model p-values, Wald test statistics for each path coefficient, and the SNP identifiers for the causal anchor sets *M*_*A *_and *M*_*B*_.

Although the main output of NEO are scores for every edge orientation, one can construct a global directed network by thresholding an edge orienting score. NEO uses the R software package *sem *[46] to compute model p-values and other fitting indices. The NEO software is documented in a series of separate tutorials that illustrate real data applications and simulation studies. These tutorials and the real data can be downloaded from our webpage.

### Applications

#### Research goals that can be addressed with NEO

NEO can be used to address the following four research goals. (1) On the simplest level, NEO can be used to assign edge orienting scores to a single edge using manually chosen genetic markers (see the example in Table 1). (2) When dealing with a single edge and multiple genetic markers, the NEO software can *automatically *select markers for edge orienting. Since the automatic marker selection entails certain parameter choices, we recommend carrying out a robustness analysis with respect to adding or removing genetic markers. (3) When dealing with a single trait *A *and manually selected genetic markers, the software can be used to screen for other traits that are causal or reactive to trait *A*. For example, in Table 2 we screen for genes that are reactive to gene expression trait *Insig1*. (4) When dealing with multiple edges, NEO can be used to arrive at a global directed network. This can be done by thresholding a chosen edge score. If the resulting global network is acyclic (i.e., it does not contain loops) then d-separation [30] and standard SEM model fitting indices can be used to evaluate the fit of the global causal model to the data.

**Table 1:** NEO analysis using manually specified genetic markers for computing edge scores.

Edge no.	Edge	LEO. NB.OCA	Cor *ρ*	Path coef	Path SE	Path Z	Model prob	Model df	*χ*^2 ^stat	RMSEA
1	rs3705921 → *Insig1*		0.22	0.18	0.081	2.2				
2	rs3670293 → *Insig1*		-0.33	-0.31	0.081	-3.8				
3	rs3675054 → *Dhcr7*		-0.26	-0.15	0.049	-3.1				
4	*Insig1 → Dhcr7*	1.2	0.81	0.79	0.049	16.1	0.24	5	6.8	0.051
5	*Insig1 → Fdft1*	1.4	0.67	0.64	0.06	10.7	0.75	5	2.7	0
6	rs3664397 → *Fdft1*		0.34	0.27	0.06	4.5				

Using the female mouse liver gene expression data, we report edge scores for the known causal relationships *Insig*1 → *Dhcr*7 and *Insig*1 → *Fdft*1 and the other edges depicted in Figure 4. The table represents a condensed summary of the NEO software spreadsheet. The high value of *LEO.NB.OCA*(*Insig*1 → *Dhcr*7) = 1.2 suggests that this causal model is 10^1.2^≈ 15.8 times more likely than the next best  local model. Similarly, *LEO.NB.OCA*(*Insig*1 → *Fdft*1) = 1.4 suggests that the causal model is 25 times more likely than the next best local model. The fourth column reports the marginal Pearson correlation coefficient, while the three path columns (standardized path coefficient, asymptotic standard error, and Z-score for the edge) give details for each individual edge in the SEM models. The last five columns summarize the fits of the two best fitting SEM models shown in Figures 4(b) and (c). The model probability column (Eq. 7) was computed using a central *χ*^2 ^statistic with the 5 degrees of freedom. The high, non-significant model p-values suggest good fit. The Root Mean Square Error of Approximation (RMSEA) is a standard SEM fit evaluation index that, similar to the *χ*^2 ^stastic, evaluates the overall fit of the SEM model; a value smaller than 0.05 is desirable.

**Table 2:** Using NEO to identify genes that are reactive to Insig1.

Edge orientation *Insig1↓*	LEO. NB. OCA female	Model prob	Path coef	Wald test pval	df	*χ*^2^	Known literature/novel (a.k.a.)	LEO. NB. OCA male	Male mice val†
*Fdft1*	1.4	0.75	0.64	<e-20	5	2.7	+	0.2	
*Dhcr7*	1.2	0.24	0.79	<e-20	5	6.8	+	1.9	*
*Scd1*	1.2	0.58	0.63	<e-20	5	3.8	+	0.4	*
*Sc4mol*	1.1	0.35	0.68	<e-20	5	5.6	+	0.5	*
*0610030G03Rik*	1.1	0.82	0.67	<e-20	5	2.2	novel (Tlcd1)	2.4	*
*Fads2*	1.0	0.64	0.61	<e-20	5	3.4	+	0.02	
*Adipor2*	0.97	0.98	0.61	<e-20	5	0.73	+	-2.0	
*Fasn*	0.96	0.72	0.77	<e-20	5	2.9	+	1.0	*
*Eaf2*	0.89	0.16	0.54	6e-16	5	8	novel, Eaf2	-0.5	
*Stard4*	0.87	0.46	0.59	<e-20	5	4.6	+	0.7	*
*Fads1*	0.86	0.82	0.73	<e-20	5	2.2	+	-0.5	
*Dlat*	0.84	0.81	0.58	<e-20	5	2.3	+	0.7	*
*Rdh11*	0.82	0.80	0.73	<e-20	5	2.3	novel, Rdh11	-0.7	
*B430110G05Rik*	0.81	0.87	0.52	2e-13	5	1.8	novel (Slc25a44)	0.7	*
*Aqp8*	0.72	0.61	0.59	<e-20	5	3.6	+	-1.6	
*Slc23a1*	0.63	0.58	0.49	1e-11	5	3.8	novel, Slc23a1	0.1	
*Slc25a1*	0.63	0.37	0.65	<e-20	5	5.4	novel, Slc25a1	-0.3	
*Acac*	0.59	0.64	0.73	<e-20	5	3.4	+	-0.4	
*Acas2*	0.58	0.19	0.64	<e-20	5	7.4	+	-2.5	
*Gale*	0.53	0.65	0.58	<e-20	5	3.3	novel, Gale	-0.3	
*Mod1*	0.38	0.60	0.59	<e-20	5	3.7	+	0.6	*
*Qdpr*	0.37	0.74	0.59	<e-20	5	2.7	novel, Qdpr	0.4	*
*6030440G05Rik*	0.35	0.70	0.54	8e-15	5	3	novel (Frmd4b)	-0.7	

Here we used the female mouse liver BxH data to illustrate that NEO can be used to identify genes that are reactive to a given trait (here *Insig1*). The table reports the 23 genes with highest *LEO.NB.OCA*(*Insig1 *→ B) scores. Since 14 of the 23 genes are already known to be reactive to *Insig1*, these results represent a highly significant validation success of NEO; using the 23388 genes on the array and assuming that there are 200 known genes downstream of Insig1 (a conservative estimate), the Fisher exact p-value of validation success is *p *= 1.0 × 10^-13^. The NEO analysis in female mice also implicates 9 novel genes. PubMed searches on these genes did not turn up any information about a role of these genes in liver or sterol homeostasis. The table also reports the analysis results using the male mice of the BxH cross. The validation (val†) column shows a star (*) if the initial finding in female liver was replicated in the independent test set of 129 F2 male mice; we defined validation success as LEO.NB.OCA score above 0.3 using the default settings of automatic SNP marker selection. The male liver analysis confirms three of the nine novel genes suggested from the female analysis: *Tlcd1, Slc25a44*, and *Qdpr*. The fact that not all genes can be replicated in the male data may reflect known differences between female and male mouse liver tissue expression profiles [48].

#### Mouse data description

We illustrate our methods using data from a previously studied F2 mouse intercross (referred to as BxH cross) [7,11,47,48] involving two inbred mouse strains (C57BL/6J.*Apoe *null and C3H/HeJ.*Apoe *null). The strain C57BL/6J is susceptible to a variety of atherosclerosis, diabetes, obesity, and heart disease related traits to which C3H/HeJ is resistant. The F2 offspring mice are expected to show a significant spectrum of atherosclerosis and metabolic syndrome responses to a high-fat diet. The mice were genotyped at 1278 genetic markers (SNPs) across the mouse genome. A variety of physiological traits were measured, including mouse body weight, fat mass, insulin, glucose, free fatty-acid levels in the blood, and cholesterol fractions (HDL and LDL+VLDL). Here we focus on gene expression data in mouse liver tissue. Since significant differences in the gene expression profiles between male and female mice have been observed [48], we analyzed each gender separately.

#### Application I: Studying the causal relationships between Insig1, Fdft1 and Dhcr7

Here we use both manually and automatically selected SNP markers to compute edge orienting scores to the known causal relationships between genes in the cholesterol biosynthesis pathways. The gene expression levels of *Insig*1 serve as a sensitive proxy for the activation level of the SREBP transcription factors [49], allowing us to study the known biology of those genes in the cholesterol biosynthesis pathway. We used the mouse liver gene expression data of the BxH mouse cross to determine whether two known causal edge orientations [50,51]*Insig*1 → *Fdft*1 and *Insig*1 → *Dhcr*7 result in high LEO.NB scores. For the female mice of the BxH cross, QTL analysis of *Insig1 *expression implicated two candidate pleiotropic anchors (SNPs) on chromosomes 8 and 16 (Figure 4a). Together these 2 SNPs explained 12.4 percent (*R*^2 ^= 0.124) of the variation of *Insig1*. As candidate orthogonal causal anchor of *Fdft1*, we selected a highly significant SNP on chromosome 9 as can be see from the single marker LOD score curve in Figure 4(a). Similarly, we found a candidate orthogonal causal anchor for *Dhcr7 *chromosome 13. Figures 4(b,c) show the causal models used to compute the model p-value in the numerators of the *LEO.NB.OCA*(*Insig*1 → *Fdft*1) score and the *LEO.NB.OCA*(*Insig*1 → *Dhcr*7) score, respectively. In Table 1, we provide more details on the edge scores of the causal models in Figure 4(b,c). We find that *LEO.NB.OCA*(*Insig*1 → *Fdft*1) = 1.4, which lies above the recommended threshold of 0.3. Further, we find that the Wald test of the path coefficient is highly significant (*Z *statistic = 10.7). The model p-value of the causal model is *p *= 0.75 and the RMSEA is ≤ 0.001. These results suggest that there is indeed a causal relationship *Insig*1 → *Fdft*1. For the edge orientation *Insig*1 → *Dhcr*7, *LEO.NB.OCA*(*Insig*1 → *Dhcr*7) = 1.2 and the Wald test is highly significant at *Z *= 16.1, and the RMSEA is 0.051. These results confirm the known causal relationship: *Insig*1 → *Dhcr*7.

For the female BxH mice, we also used automatic SNP selection to compute LEO.NB scores. For edge orientations *Insig*1 → *Dhcr*7 and *Insig*1 → *Fdft*1, the results of a robustness analysis are presented in Figures 5(a) and 5(b), respectively. The robustness analysis suggests that both edges are causal in female mice since the LEO.NB.CPA scores remain above the recommended threshold of 0.8. However, the robustness analysis of LEO.NB.OCA for edge *Insig*1 → *Fdft*1 (Figure 5b) shows that for a particular set of automatically selected markers, the score dips below the recommended threshold of 0.3 for this score. Since automatic SNP selection is particularly vulnerable to false positive causal anchors, it is advisable to replicate the NEO analysis in an independent data set. For example, we also used automatic SNP selection to compute edge orienting scores in male mice of the BxH cross. Although causal relationships may differ between male and female mice, replication in male mice certainly provides evidence that the reported causal relationships are true. Figures 5(c) and 5(d) show the results of a robustness analysis for *LEO.NB.OCA*(*Insig*1 → *Dhcr*7) and *LEO.NB.OCA*(*Insig*1 → *Fdft*1), respectively. Overall, we find that automatic marker selection with the LEO.NB.OCA and LEO.NB.CPA scores provide evidence of the reported causal relationships in both male and female mouse liver data.

#### Application II: Screening for genes that are reactive to Insig1

In this application, we illustrate that for a single trait (here *Insig1*) and manually selected genetic markers NEO can be used to screen for other traits that are reactive to the trait in question.

We again used the above-mentioned genetic markers on chromosomes 8 and 16 as causal anchors for *Insig1*. For each gene expression trait *B*, we computed a LEO.NB score for the edge *Insig1 *→ *B*. Table 2 reports details for 23 highest ranking genes. Prior literature [50,51] suggests that 14 out of the 23 genes are reactive to *Insig1 *and are part of the well-studied sterol homeostasis pathway. Since so many known sterol regulated positive controls are recovered simultaneously, these findings are highly significant. Using the 23388 array genes (probes), and assuming that there are 200 known genes downstream of *Insig*1 (a conservative estimate), we compute the Fisher exact test for the set of 9 predicted versus 14 known downstream genes giving a p-value of 1.0 × 10^-13 ^for the predicted novel gene set.

Moreover, our analysis also implicates nine novel genes as being affected by the same pathway in female liver. A PubMed literature search on these genes did not suggest known relationships to liver or sterol impacted gene expression.

NEO gene screening requires careful validation. For example, we report the results of a NEO analysis in male mouse liver data in Table 2. Of the nine novel genes suggested from the female analysis, the male liver analysis confirms three of these: *Tlcd1*, *Slc25a44*, and *Qdpr*. The disparity between male and female mice may reflect the tissue-specific expression and regulation of sexually dimorphic genes [52].

#### Relationship to prior work

The above application describes the use of NEO for finding reactive genes to *Insig*1. Here we contrast this NEO based gene screening method to a related gene screening method [15] that utilizes a hybrid between the single anchor and the candidate pleiotropic anchor approach (refer to our Figure 1, panels (a) and (b)). Similar to our computation of the LEO.NB.CPA score, the authors use a forward-backward stepwise regression procedure to build the initial genetic model for the downstream trait. For each locus retained in the genetic model for a given trait, their LCMS (Likelihood-based Causality Model Selection) test evaluates genes for causality by comparing three of the five single anchor models (models 1, 2, and 3) shown in our Figure 2(a) for smallest AIC. Taking just the genes for which the causal model – model 1 of Figure 2(a) for a single gene A and trait B – fits best for at least two common pleiotropic markers, the candidate causal gene list is generated by ranking genes according to 'the amount of genetic variance of the trait that was causally explained by variation in their transcript abundance,' which amounts to comparing the p-values of the CPA models for all final candidate genes. In contrast, NEO makes use of orthogonal anchors and fits multiple orthogonal anchors simultaneously. Our simulations suggest power advantages of the resulting LEO.NB.OCA score (Additional Figure 1).

#### Application III: Fsp27 is upstream of a biologically interesting gene co-expression module in female BxH mice

Here we illustrate how NEO can be used to assign edge orienting scores to a single edge using manually and automatically chosen genetic markers. Specifically, we computed edge orienting scores for the edge *Fsp*27 → *MEblue *where *Fsp27 *(also known as Cidec) corresponds to a pro-apoptotic gene that is related to metabolic syndrome: *Fsp27*-null mice have been found to be resistant to obesity and diabetes; *Fsp27 *expression is halved in obese humans after weight loss; and *Fsp27 *regulates lipolysis in white human adipocytes [53]. The other quantitative trait, *MEblue*, represents the activation status of an entire pathway. More specifically, *MEblue *is the module eigengene (i.e., the first principal component) of the biologically important 'blue' gene co-expression module described in [7,11]. This gene co-expression module was comprised of highly correlated genes and *MEblue *is a summary gene expression trait that best represents the expressions of the blue module genes.

To study whether *Fsp27 *causally affects *MEblue*, we conducted both manual and automatic SNP selection approaches. We used a previously identified SNP marker on chromosome 19 (SNP19) that affected the expression of the blue module genes [7,11] and of several physiologic traits as the manually chosen input SNP. This genetic marker was previously referred to as a module quantitative trait locus (mQTL) since it was found to affect the gene expression profiles of most blue module genes. Using this SNP, we found highly causal LEO.NB scores between *Fsp27 *and *MEblue*. The LEO.NB.OCA scores passed the threshold of 0.3 and the LEO.NB.CPA scores passed the threshold of 0.8. We also used the automatic SNP selection strategies to assess the causal relationship between *Fsp27 *and *MEblue*. The results of a robustness analysis can be found in Figure 6. We find that the causal relationship *Fsp*27 → *MEblue *is highly robust with respect to different automatic marker selection methods.

### Simulation studies

#### Multi-edge simulation model that involves one hundred SNPs

NEO analysis can orient the edges of a multi-trait network by automatically selecting markers for each trait separately. The analyses proceed in a stepwise fashion: edges are oriented one at the time. For each edge, NEO computes edge orienting scores (LEO.NB.OCA, LEO.NB.CPA, etc). By thresholding these edge orienting scores, one can arrive at a globally oriented trait network. The details of the simulation model and relevant R code is presented in an R software tutorial on our webpage. Briefly, we simulated a causal network between five gene expressions (denoted by *E*1 through *E*5) and a trait (denoted by *Trait*).

Each of the 6 traits was simulated to be under the causal influence of 3 SNPs. We added 82 noise SNPs so that the data contained 100 SNPs.

We simulated the following causal relationships between the traits:

*E*1 → *E*2

*E*1 → *E*3

*E*3 ← *HiddenConfounder *→ *E*4

*E*4 → *Trait*

*Trait *→ *E*5.

Note that the correlation between traits *E*3 and *E*4 was entirely due a hidden confounder. The heatmap plot in Figure 7(a) depicts the true causal model. Note that a red square in the i-th row and j-th column indicates that trait *i *causally affects trait *j*. The rows and columns of the heatmap are ordered according to a hierarchical clustering tree, which was constructed using average linkage hierarchical clustering with the dissimilarity *diss*(*Ei*, *Ej*) = 1 - |*cor*(*Ei*, *Ej*)|. Figure 7(b) shows the corresponding heatmap of the observed network that was reconstructed using the LEO.NB.OCA score. Figure 7(c) shows an alternative output graph of NEO. Blue edges indicate significant correlations (at a user-supplied threshold) and a LEO.NB.OCA score is added to each edges whose LEO.NB.OCA score passes a user-supplied threshold. We find that all true causal edges are correctly retrieved at the recommended LEO.NB.OCA threshold of 0.3. Figure 7(d) shows the results of a robustness analysis for the LEO.NB.OCA and LEO.NB.CPA scores for the edge orientation *E*4 → *Trait*. The LEO.NB.OCA and the LEO.NB.CPA scores exceed their respective threshold of 0.3 and 0.8 for all steps of the robustness analysis, i.e., they retrieve the orientation correctly. Alternative simulation models can be explored using our online tutorial.

#### Single edge simulation model parameterized with the heritability

In Additional File 1, we describe several simulation studies that use a single edge simulation model. Briefly, we simulated two traits *A *and *B *that are anchored to genetic marker sets *M*_*A *_and *M*_*B*_, respectively. The correlation *cor*(*A*, *B*) results from both a causal influence of *B *on *A *and from a hidden confounder *C*. This single edge model is used i) to study the choice of thresholds for the LEO.NB scores, ii) to compare the LEO.NB.CPA with LEO.NB.OCA scores, and iii) to evaluate automatic SNP selection methods. The results of these simulations are described in Additional File 1 and in our online R software tutorials.

## Discussion

We propose methods for using multiple genetic markers to recover causal trait-trait relationships in systems genetic studies. NEO will be particularly useful for the analysis of experiments in which common genetic variations are leveraged to explore complex genetic traits.

We propose several edge orienting scores that measure the genetic evidence in favor of a given edge orientation *A *→ *B*. While several methods exist for constructing undirected gene co-expression networks based on thousands of genes, we have evaluated the NEO method for inferring directed networks involving relatively few genes (fewer than 10 in our simulations). Future research could explore the use of the method for inferring directed networks involving thousands of genes.

Our simulation studies show that orthogonal causal anchors lead to powerful edge scores that may outperform scores based only on candidate pleiotropic anchors (Additional Figure 1 in Additional File 1). To afford flexibility to the user, the NEO software provides several options for anchoring the traits to genetic markers (manual versus automatic), computing local edge scores (LEO.NB.CPA, LEO.OCA), and diagnosing poor model fit (RMSEA, CFI score, etc). NEO provides multiple options for automatically anchoring a trait to genetic markers: greedy, forward, and combined (greedy and forward) SNP marker selection. While our simulation studies suggest that these three SNP marker selection methods have similar performance, we find that the combined SNP marker selection performs best when signal SNPs are in high linkage disequilibrium with noise SNPs (Figure 2 in Additional File 1).

NEO's local, stepwise approach for orienting edges of a trait network allows one to orient networks involving hundreds or even thousands of traits. Since the calculation of edge orienting scores is based on local causal models, NEO is relatively robust with regard to mistaken orientation of some edges in the global network.

Although NEO performs well in simulation studies and the reported real data applications, we note that it has several limitations. The first limitation is that it requires the availability of genetic markers that are significantly associated with at least one trait per edge. Spurious associations between the markers and traits will result in meaningless edge orienting scores. Although the multi-marker score (LEO.NB.OCA) is quite robust to noise SNPs in our simulations, false-positive input SNPs will result in unreliable edge scores. The automatic SNP selection is particularly vulnerable to false positives and its results should be carefully validated using biological experiments or causality analysis of independent data.

The second limitation is that the resulting global trait network may contain loops, i.e. it may be cyclic. In contrast, a directed acyclic graph (DAG) has no cycles. DAGs appear in models where it does not make sense for a trait to have a path to itself. While local DAGs are used for orienting individual edges, the reconstructed global trait network may no longer be acyclic. Acyclicity is theoretically desirable since it allows one to test causal predictions using Pearl's formalism of d-separation [30-32]. The constraint of acyclic graphs in many network learning algorithms is often more a mathematical convenience than reflective of biology; cycles may reflect feedback loops for maintaining homeostasis. When more and more edges are oriented, as in the IC/IC* [31] and PC/PC* [54] algorithms, an error in one part of the network can propagate and cause erroneous orientations in unrelated portions of the network. Most often these errors arise due to confusion between confounded and truly causal flows. To avoid being misled, NEO deliberately discards the evidence from correlated trait neighbors in the undirected graph during LEO scoring. By computing local edge orienting scores without regard to a global acyclicity constraint, the analysis is relatively robust to mis-oriented neighboring edges. NEO uses causal anchors for each edge separately and thus allows the genetic data to speak for themselves.

The proposed LEO.NB scores are local in that they orient one edge at a time without regard to the orientations of the other edges. The reconstruction of the global network should be taken with a grain of salt. While we report one simulation model where the global network was reconstructed correctly, future research should carefully evaluate the performance of the NEO approach for inferring global networks. A potential use of NEO is to use it for initializing an iterative edge orienting algorithms for large networks that maximizes a global SEM fitting index.

The third limitation is that the SEM-based edge orienting scores assume linear relationships between traits and SNP markers. This is mathematically convenient but non-linear effects are common and have been reported in the literature [55]. The NEO approach works in the domain of linear graphical models since it is based on correlations and SEMs. Akin to the use of Pearson versus Spearman correlation, the software also offers the option of modelling monotone quantitative relationships in NEO by converting all data to ranks before further processing. NEO will not work for traits that satisfy non-monotone relationships. A fourth limitation is that the influence of genetic markers may be indirect. NEO may miss some relationships. While SNP changes must be upstream (causal) of gene expression and phenotype manifestations, this does not preclude some SNPs from modifying the action of other SNPs, and the effect of such modifiers may become apparent only in particular contexts.

Causal inference and structural equation modeling assume that relevant traits and causal anchors have been included in the causal model. Under-specified causal models, i.e. models that omit important variables, may mislead the user to detect spurious causal relationships. NEO leads to relatively simple causal networks that do not incorporate dynamic or hierarchical properties (compare to [56-58]). Given all these potential limitations it is reassuring that NEO performs well at retrieving known causal relationships in the reported real data applications. Since NEO focuses on individual edges, we expect that NEO will be particularly useful for identifying traits that are causal for (or reactive to) a given trait. For example, we illustrate that NEO can be used to identify gene expression traits that are reactive to *Insig*1. The *sem *R package can be used to evaluate the global fit of an acyclic multi-trait network.

The NEO algorithm computes an edge score for each edge without regard to the information gained from neighboring edges. NEO aims to harness the power of the established upstream causal anchors (markers) as fully as possible; thus, it is appropriate when genetic variations are a major source of the variation in the traits. To the extent the environment (e.g. diet) is also varied, the NEO approach may be less effective. It is plausible that additional assumptions may allow one to use unshielded colliders to improve the causal inference [32]. This is a promising avenue of future research.

We focused on the use of SNP markers which capture only a limited amount of the sequence information of each individual. In the not too distant future, it will be economically feasible to obtain the sequence information of each study subject. Since sequence information is likely to enhance the causal anchor assignment, sequence data may greatly improve the power of the NEO method. Apart from the common genetic variation that perturbs gene expression in mouse crosses, NEO can also be applied to orient edges on the basis of causal anchors from population-based allelic association studies, cell hybrids, or transfected cells.

## Conclusion

Natural randomization of alleles that occurs during meiosis can be used to study the causal information flow through trait networks. For example, we use mouse cross data to retrieve known causal relationships in the sterol biosynthesis pathway. We find that the proposed edge scores (LEO.NB.OCA, LEO.NB.CPA) are quite robust with respect to adding extraneous noise SNPs. Combined with the use of orthogonal causal anchors, the proposed edge orienting scores can provide a strong basis for further experimental evaluation of the predicted causal relationships.

## Methods

A detailed description of our methods, the data, and the R software scripts can be downloaded from our webpage. Here we will briefly outline the main points.

### Review of Structural Equation Models

Structural equation modelling descends from Sewall Wright's path analysis and is a generalization of multivariate linear regression analysis. Since maximum likelihood testing procedures were incorporated into the analysis, SEMs have become a widely used tool to explore the causal relationship between multiple variables [31,32,45,46,59,60]. Structural equation modelling has also been found useful for describing the relationships between traits and genetic markers [17].

SEM analysis typically starts with variables centered on their means and focuses on the covariance relationships. Traits or nodes are connected by arrows denoting causal relationships. The causal relationships define a systems of linear regression models where the parents of a node are used to predict the child node's response. The system of resulting linear equations imposes constraints on the structure of the expected covariance matrix. Given *m *observed traits, we denote the observed sample covariance matrix by *S*_*m *× *m *_and the expected covariance matrix under the causal model by Σ(*θ*). For the models considered in this article, the parameters *θ *include path coefficients between the traits and variances of the genetic markers. To arrive at a maximum likelihood estimate of the model covariance matrix $\hat{\Sigma }=\Sigma (\hat{\theta })$, the following statistical criterion is minimized:

*F*(*θ*) = ln |(Σ*θ*)| + *tr*(*S*Σ^-1^(*θ*)) - *m *- ln |*S*|

We denote the maximum likelihood estimate of *θ *by $\hat{\theta }$ and the corresponding maximum likelihood by *F*($\hat{\theta }$). The SEM model chi-square statistic is defined as follows:

(7) $$X^{2}=(N-1)F(\hat{\theta })$$

where *N *denotes the sample size. The null hypothesis states that the expected covariance matrix equals that of the underlying causal model. In large samples and assuming multivariate normality, *X*^2 ^is distributed as a Pearson chi-square statistic. This statistic is known as the model chi-square or generalized likelihood ratio statistic. If *X*^2 ^= 0, the causal model perfectly fits the data. If the causal model is correct then *X*^2 ^asymptotically follows a central chi-square distribution $X^{2}\sim \chi^{2}(df=\frac{m(m+1)}{2}-t)$ with degrees of freedom *df *determined by the number of observed variables *m *and the number of free parameters *t*. The model chi-square statistic statistic *X*^2 ^can be used to compute a model p-value for each causal model. For example, *P*(*M *→ *A *→ *B*) = *P*(*data*|*M *→ *A *→ *B*) denotes the p-value for the model in which SNP marker *M *causally affects trait *A *which in turn affects trait *B*. *X*^2 ^tests the null hypothesis that the model is correct. A small model p-value (say *p *< 0.05) indicates that the causal model does not fit well. Following the logic of an 'accept-support' context [59,61] where the null hypothesis represents the researchers belief, it is the failure to reject the null hypothesis that supports the causal model.

The *X*^2 ^fit statistic and the corresponding model p-value have several limitations, e.g. they are sensitive to the size of correlations and they depend on the sample size *N *[59]. Despite these limitations, we chose the model p-value as the basis of the LEO.NB scores (Eq. 4) because it is the key ingredient of most, if not all, alternative fitting indices. Our model p-value based LEO.NB score can be considered as a *relative *fitting index that contrasts the fit of the causal orientation to that of the other models. Alternative edge orienting scores could be defined by replacing the model p-value by another fitting index for which high values indicate good fit, e.g. the comparative fitting index (CFI). Studying the performance of these generalizations of the LEO.NB score is beyond the scope of this article.

## Availability and requirements

Project name: Network Edge Orienting (NEO) R software

Project home page: 

Operating system(s): Platform independent

Programming language: R

Licence: GNU GPL 3

## Authors' contributions

JEA and SH jointly developed the methods and wrote the article. JEA implemented the NEO software. TFF evaluated the method in several real data applications, helped with the R software tutorials, and the write-up. SH and AJL directed the methodological research and applications, respectively. All authors read and approved the final manuscript.

## Supplementary Material

Additional file 1 **Single edge simulation study**. This document describes our single edge simulation studies involving the LEO.NB.CPA score (Eq. 5) and the LEO.NB.OCA score (Eq. 6). We describe the parameters used in the single edge *A *← *B *simulation model. A hidden confounder *C *affects the correlation between *A *and *B*. The effect of SNP markers on traits *A *and *B *is parameterized with the restricted heritabilities. The single edge simulation model is used i) to study the choice of thresholds for the LEO.NB scores, ii) to compare the LEO.NB.CPA with LEO.NB.OCA scores, and iii) to evaluate automatic SNP selection methods. Click here for file
