# Supplementary material for: Using genetic markers to orient the edges in quantitative trait networks: The NEO software
Source: BMC Syst Biol. 2008 Apr 15;2:34. doi: 10.1186/1752-0509-2-34 (PMC2387136; doi:10.1186/1752-0509-2-34)
Supplement: Additional file 1 — Single edge simulation study. This document describes our single edge simulation studies involving the LEO.NB.CPA score (Eq. 5) and the LEO.NB.OCA score (Eq. 6). We describe the parameters used in the single edge A ← B simulation model. A hidden confounder C affects the correlation between A and B. The effect of SNP markers on traits A and B is parameterized with the restricted heritabilities. The single edge simulation model is used i) to study the choice of thresholds for the LEO.NB scores, ii) to compare the LEO.NB.CPA with LEO.NB.OCA scores, and iii) to evaluate automatic SNP selection methods. [file 1752-0509-2-34-S1.pdf]

Additional File 1: Single Edge Simulations for Evaluating the NEO software

Jason E. Aten[1,2], Tova F. Fuller[1], Aldons J. Lusis[1,3], Steve Horvath*[1,4]

[1] Human Genetics, David Geffen School of Medicine University of California, Los Angeles, CA 90095, USA [2] Biomathematics, UCLA [3] Microbiology, Immunology and Molecular Genetics, UCLA [4] Biostatistics, School of Public Health, UCLA

Email: Jason E. Aten - jaten@ucla.edu; Tova Fuller - suprtova@ucla.edu; Aldons J. Lusis - jlusis@mednet.ucla.edu; Steve Horvath*- shorvath@mednet.ucla.edu;

*Corresponding author

Abstract

In this Additional File, we describe a simple simulation model for studying the effect of genetic markers and a hidden confounding variable C on two observed traits A and B. The model uses the correlation $cor(A, B)$ between traits A and B as input parameter. The observed correlation $cor(A, B)$ results from two sources: the direct causal influence of B on A and a shared confounding variable C.

Background

We briefly review our definitions of the edge orienting scores before describing our simulation results. To summarize the genetic evidence in favor of a given edge orientation $A \to B$, we propose the use of edge orienting scores. The higher the value of an edge orienting score for orientation $A \to B$, the stronger the

genetic evidence favors this causal model. We define the single-marker LEO.NB score as follows:

$$LEO.NB.SingleMarker(A \rightarrow B|M) = \log_{10}\left(\frac{P(\text{model 1: } M \rightarrow A \rightarrow B)}{\max\begin{pmatrix} P(\text{model 2: } M \rightarrow B \rightarrow A), \\ P(\text{model 3: } A \leftarrow M \rightarrow B), \\ P(\text{model 4: } M \rightarrow A \leftarrow B), \\ P(\text{model 5: } M \rightarrow B \leftarrow A) \end{pmatrix}}\right). \quad (1)$$

A positive $LEO.NB(A \rightarrow B)$ score indicates that the p-value in favor of model $A \rightarrow B$ is higher than that of any of the competing models. A negative LEO.NB score indicates that the $A \rightarrow B$ model is inferior to at least one alternative model. We suggest to use a LEO.NB.SingleMarker threshold of 1 which implies that the causal model in the numerator has a $10 = 10^1$ fold higher p-value than the next best model in the denominator.

Multi-marker LEO.NB score

It is straightforward to generalize the single marker LEO.NB score (Eq. 1) to a set of genetic markers $M_A = \{M_A^{(1)}, M_A^{(2)}, \ldots\}$:

$$LEO.NB.CPA(A \rightarrow B|M_A) = \log_{10}\left(\frac{P(\text{model 1: } M_A \rightarrow A \rightarrow B)}{\max\begin{pmatrix} P(\text{model 2: } M_A \rightarrow B \rightarrow A), \\ P(\text{model 3: } A \leftarrow M_A \rightarrow B), \\ P(\text{model 4: } M_A \rightarrow A \leftarrow B), \\ P(\text{model 5: } M_A \rightarrow B \leftarrow A) \end{pmatrix}}\right). \quad (2)$$

If an additional genetic marker set $M_B = \{M_B^{(1)}, M_B^{(2)}, \ldots M_B^{(K_B)}\}$ associated with trait B is available, it allows for the definition of a likelihood-based orthogonal causal anchor (OCA) score which assesses whether the model $M_A \rightarrow A \rightarrow B \leftarrow M_B$ has a higher p-value than the alternative models. Specifically, we define

$$LEO.NB.OCA(A \rightarrow B|M_A, M_B) = \log_{10}\left(\frac{P(\text{model 1: } M_A \rightarrow A \rightarrow B \leftarrow M_B)}{\max\begin{pmatrix} P(\text{model 2: } M_A \rightarrow A \leftarrow B \leftarrow M_B), \\ P(\text{model 3: } B \leftarrow M_A \rightarrow A; A \leftarrow M_B \rightarrow B), \\ P(\text{model 4: } M_A \rightarrow A \leftarrow C \rightarrow B \leftarrow M_B) \end{pmatrix}}\right). \quad (3)$$

Single edge simulation model parameterized by heritability

Here we describe a simple simulation model for studying the effect of genetic markers and a hidden confounding variable C on two observed traits A and B. The model, which is depicted in Figure 1(a), uses the correlation $cor(A,B)$ between traits A and B as input parameter. The observed correlation $cor(A,B)$ results from two sources: the direct causal influence of B on A (determined by the path coefficient ω) and a shared confounding variable C (determined by the path coefficients γ). We assume that trait B is determined by genetic markers $M_B^{(j)}$, the confounder C and measurement noise ϵ_B. Trait A is determined by genetic markers $M_A^{(i)}$, the confounder C and, trait B and measurement noise ϵ_A:

$$\begin{aligned} A &= \sum_i \alpha_i M_A^{(i)} + \gamma C + \omega B + \epsilon_A \\ B &= \sum_j \beta_j M_B^{(j)} + \gamma C + \epsilon_B \\ C &\sim N(0,1) \\ \epsilon_A &\sim N(0, \sigma^2_{\epsilon_A}) \\ \epsilon_B &\sim N(0, \sigma^2_{\epsilon_B}). \end{aligned}$$

We assume that the variances of all traits and all genetic markers are equal to 1,
i.e. $Var(A) = Var(B) = Var(C) = Var(M_A^{(i)}) = Var(M_B^{(j)}) = 1$. To determine the effect of the genetic markers on traits A and B, we use the heritability as parameter since it is intuitive to geneticists. The genetic heritability measures the proportion of trait variation that is due to underlying genetic variation. For traits A and B, we define the restricted heritabilities h'_A of h'_B, respectively, as follows

$$h'_A = \frac{Var(\sum_i \alpha_i M_A^{(i)})}{Var(A)} \tag{4}$$

$$h'_B = \frac{Var(\sum_j \beta_j M_B^{(j)})}{Var(B)}. \tag{5}$$

Then one can easily show that $h'_A = \sum \alpha_i^2$, $h'_B = \sum \beta_i^2$, and that $0 \leq h'_A, h'_B < 1$. As an aside, we mention that the restricted heritability of B equals the unrestricted heritability. By contrast, the restricted heritability of A is smaller than the unrestricted heritability, since it does not take account of the genetic markers $M_B^{(j)}$ that are acting through B.

We also assume that the markers influencing A and the markers influencing B are independent conditional upon A and B. Under these assumptions, the variances and covariances of the standardized variables A, B,

and C are as follows:

$$\begin{aligned}
\text{Var}(A) &= h'_A + \gamma^2 + \omega^2 + 2\gamma^2\omega + \sigma^2_{\epsilon_A} = 1 \\
\text{Var}(B) &= h'_B + \gamma^2 + \sigma^2_{\epsilon_B} = 1 \\
\text{Cov}(A,B) &= \gamma^2 + \omega \\
\text{Cov}(A,C) &= \gamma + \omega\gamma \\
\text{Cov}(B,C) &= \gamma
\end{aligned}$$

Note that the correlation between A and B is given by $\rho_{AB} = \text{Cor}(A,B) = \text{Cov}(A,B) = \omega + \gamma^2$, i.e. the correlation ρ_{AB} is the sum of a causal part ω and a confounded part γ^2. Therefore, we define the proportion of the causal signal θ_{causal} in correlation ρ_{AB} as follows:

$$\theta_{causal} = \omega/(\omega + \gamma^2). \tag{6}$$

Our simulations use the following input parameters: ρ_{AB}, θ_{causal}, h'_A, h'_B, the number of genetic markers K_A and K_B, and the number of observations N. The other parameters can be computed from these input parameters, e.g.

$$\begin{aligned}
\omega &= \rho_{AB} \cdot \theta_{causal} \\
\sigma^2_{\epsilon_A} &= 1 - 2\omega\gamma^2 - \gamma^2 - \omega^2 - h'_A \\
\sigma^2_{\epsilon_B} &= 1 - \gamma^2 - h'_B.
\end{aligned}$$

More details on the simulation model are provided in an R software tutorial on our webpage:
www.genetics.ucla.edu/labs/horvath/CoexpressionNetwork/NEO.

Single edge simulation model parameterized with the heritability

In the following, we describe several simulation studies that use the single edge simulation model depicted in Figure 1(a). A detailed description of the single edge simulation model can be found in the Methods section and in an online R software tutorial. Briefly, we simulated two traits, A and B, anchored to genetic marker sets M_A and M_B, respectively. The correlation $cor(A,B)$ results from a causal influence of B on A and from a hidden confounder C. We denote by θ_{causal} the proportion of true causal signal in the correlation $cor(A,B) = \rho_{A,B}$. The restricted heritability h'_A is defined as proportion of variance of A due to variation in the genetic marker set M_A. Analogously, we define the restricted heritability h'_B of trait B.

Choosing thresholds for the LEO.NB scores

Here we use simulation studies to study the tradeoff between false positive rates and power for the multi-marker LEO.NB scores.

We recommend that the user choose a threshold of 0.3 for the LEO.NB.OCA score and a threshold of 0.8 for the LEO.NB.CPA score. Thresholds of 0.3 and 0.8 imply that the causal model (in the numerator of the LEO.NB score) have a $2 = 10^{0.3}$ and $6.3 = 10^{0.3}$ fold higher p-value, respectively, than the next best model in the denominator.

The thresholds leads to different false positive rates and powers depending on the underlying causal model. To illustrate this point, we report the results of different simulation studies in Table 1. We used the single edge simulation model (Figure 1a) to evaluate the LEO.NB.OCA score (Eq. 3) and the LEO.NB.CPA score (Eq. 2) with respect to retrieving the causal orientation $A \leftarrow B$. We simulated causal anchors $M_A^{(i)}$ and $M_B^{(j)}$ for traits A and B, respectively. The restricted heritabilities (Eq. 4) of both traits were set to $h'_A = h'_B = 0.4$. We kept the correlation fixed ($cor(A, B) = \rho_{A,B} = 0.3$) but varied the proportion of true causal signal, θ_{causal} (Eq. 6). When the causal signal, θ_{causal}, is 0 the correlation between A and B stems from the unobserved confounder C. We simulated $N = 200$ observations and 100 background noise SNPs that were unrelated to the traits.

In Table 1, we report the results of four different causal models for the edge $B \rightarrow A$. Simulation models '1a.1b.0', '1a.1b.50', and '1a.1b.100' involved one signal SNP into A, one signal SNP into B and varying the proportion of causal signal with θ_{causal} set to 0, 0.5, and 1.0, respectively. Simulation model '1a.1b.100.4an.4bn' also had a causal signal of $\theta_{causal} = 1.00$, but it involved four neighboring noise SNPs into A and four into B. The correlations between the neighboring noise SNPs and the primary signal SNP were randomly sampled from $[0.4, 0.7]$. Thus, model 1a.1b.100.4an.4bn allows one to study how noise SNPs that are in high linkage disequilibrium with signal SNPs affect the computation of edge orienting scores. For each simulation model, we simulated 300 replicate data sets and report the average power and false positive rate in Table 1. The null model 1a.1b.0 allows us to estimate the false positive rate of the LEO.NB scores. At a threshold of 0.3, the LEO.NB.OCA score and the LEO.NB.CPA score have a false positive rate of 0.0041 and 0.027, respectively. Thus, the CPA score is less conservative than the OCA score, which is why we recommend a higher threshold of 0.8 for the CPA score.

The single signal marker results in Table 1 show that when the false positive rate is kept at or below 5%, the LEO.NB.OCA score has high power to retrieve the causal signal. Moreover, at the recommended threshold of 0.3, the LEO.NB.OCA score maintains a false positive rate below 1% while simultaneously

obtaining statistical power at or above 90%.

For the model 1a.1b.0, we find empirically that a 5% false positive rate corresponds to a threshold of -1.61 for LEO.NB.OCA and -0.2 for LEO.NB.CPA. Since the LEO.NB threshold of 0.3 is less conservative for the CPA score than for the OCA score, it is not surprising that for the semi-causal model 1a.1b.50 the power of the CPA score (0.38) is slightly higher than that of the OCA score (0.29). However, under the fully causal model 1a.1b.100, the power of the CPA score (0.83) is slightly lower than that of the OCA score (0.90).

To compare the LEO.NB.CPA and the LEO.NB.OCA scores at the same false positive rate, a LEO.NB.CPA threshold of 1.552 would be needed to obtain the false positive rate of 0.0041 (± 0.00078 SE). At this threshold, the LEO.NB.CPA score would yield only 48% (± 2.3% SE) power for the fully causal model (1a.1b.100causal) while the corresponding power of the OCA score was 0.94. These results show that the LEO.NB.OCA score can be far superior to the LEO.NB.CPA score.

Single edge model for comparing LEO.NB.CPA with LEO.NB.OCA

Here we use the single edge simulation model (Figure 1a) to compare the power of the LEO.NB.OCA score (Eq. 3) with that of the LEO.NB.CPA score (Eq. 2) with respect to retrieving the causal orientation $A \leftarrow B$. We simulated causal anchors $M_A^{(1)}$ and $M_B^{(1)}$ for traits A and B, respectively. We kept the correlation fixed($cor(A, B) = \rho_{A,B} = 0.4$), but varied the proportion of true causal signal θ_{causal} (Eq. 6). We simulated $N = 200$ observations. Figures 1 (b) and (c) correspond to models with restricted heritabilities $h'_A = h'_B = 0.2$ and $h'_A = h'_B = 0.6$, respectively. To allow for a fair comparison, we chose thresholds that result in comparable false positive rates for both scores. As the proportion of causal signal θ_{causal} increases (and the extent of confounding γ^2 lessens in turn), the power of both edge orienting scores increases. As the signal to noise ratio passes the 1:1 threshold ($\theta_{causal} = 0.50$ causal signal), the power of the scores goes to 70% and then 80%, with the orthomarker scores showing slightly better performance (in Figure 1c). Since the LEO.NB.OCA score includes the genetic information present in the orthogonal causal anchor set M_B, it is not surprising that it tends to have higher power than the LEO.NB.CPA score in this example.

The $LEO.NB.OCA(A \leftarrow B)$ score is expected to be inferior to the $LEO.NB.CPA(A \leftarrow B)$ score if trait A is not associated with a SNP since then the OCA score may erroneously anchor trait A to one or more noise SNPs. To illustrate this, we simulated the fully causal simulation models as described above but did not anchor A to a genetic marker, i.e. $h'_A = 0$. At a LEO.NB.OCA threshold of 0.3, we found that the

LEO.NB.OCA score has a mean false positive rate of 0.0031 (Standard Error $SE = 0.0006$) and a mean power of 0.93 ($SE = 0.008$). At this threshold, the LEO.NB.CPA has a smaller false positive rate of 0.0018 ($SE = 0.0003$) and a higher power 0.95 ($SE = 0.006$). We should point out that at the recommended threshold of 0.8 for the CPA score, the this score would have a smaller false positive rate but also a lower power.

Simulations to evaluate automatic SNP selection methods

NEO software implements several options for automatically selecting SNPs: a greedy approach, a forward-stepwise approach or both. Here we evaluate the performance of these selection methods and their robustness with respect to noise SNPs. We use the single edge simulation model (Figure 1a). For the two traits A and B, we simulated an observed correlation of $cor(A,B) = \rho_{A,B} = 0.3$, and restricted heritabilities $h'_A = h'_B = 0.4$. We simulated a fully causal model ($\theta_{causal} = 1.0$) and $N = 200$ observations. In this simulation, we include neighboring noise SNPs that are correlated (in high linkage disequilibrium) with the primary signal SNPs, albeit with a weaker trait correlation. Each of these neighboring SNPs had a correlation sampled uniformly from $[0.4, 0.7]$ with their respective primary causal SNPs.

Figures 2(a,b) correspond to the situation where each trait is anchored to one true signal SNP. Figures 2(c,d) involve traits related to four true SNPs. We find that both LEO.NB scores peak at the true number of signal SNPs. The LEO.NB.OCA scores pass the recommended threshold of 0.3 even when only a single signal SNP is used. As expected, the scores increase as additional signal SNPs are added. It is reassuring that the scores are quite robust with respect to extraneous noise SNPs.

When comparing the results of the three automatic SNP selection approaches, we find that the LEO.NB scores are mildly susceptible to the decoy effect of neighboring (high LD) noise SNPs if only greedy SNP selection is used to define the preliminary sets M'_A (Step 2). Fortunately, this effect is ameliorated by the combined use of greedy and forward-step automatic SNP selection procedures. Since neighboring SNPs are increasingly common as whole genome SNP data become available, our simulation study shows that a strictly greedy approach should be supplemented by a forward-step approach to increase the power of edge orienting.

In Figures 2(e,f) we show the null distribution of the scores under the scenario of 0% causal signal. These figures extend the results of Table 1 by varying the number of SNPs that are used in the computation of the LEO.NB scores. When the correlation between A and B is due to a shared yet unobserved or hidden causal parent C ($M_A \rightarrow A \leftarrow C \rightarrow B \leftarrow M_B$) rather than true $A \rightarrow B$ or $B \rightarrow A$ causal flow, the

LEO.NB.OCA score – which includes a confounded model in the denominator – stays substantially below its recommnended threshold of 0.3. This study highlights an advantage of the proposed LEO.NB.OCA score: it is conservative and robust irrespective of the automatic marker selection method.

Discussion

Our simulation studies show that orthogonal causal anchors lead to powerful edge scores that may outperform scores based only on common pleiotropic anchors (Figure 1).

NEO provides multiple options for automatically anchoring a trait to genetic markers: greedy, forward, and combined (greedy and forward) SNP marker selection. While our simulation studies suggest that these three SNP marker selection methods have similar performance, we find that the combined SNP marker selection performs best when signal SNPs are in high linkage disequilibrium with noise SNPs. We find that the proposed edge scores (LEO.NB.OCA, LEO.NB.CPA) are quite robust with respect to adding extraneous noise SNPs.

Our simulations do not evaluate NEO by considering all steps jointly (how to best construct the undirected network, how to best identify marker/QTL sets for each expression trait, followed by edge orientation). Instead, the simulations evaluate the power and false positive rate given the described marker assignment. Errors in the specifications of the edges or the causal anchor assignment will greatly inflate the reported false positive rates. Although presented for interest and as a starting point for further exploration, the automated SNP selection methodology is not yet amenable to evaluation by theoretical or permutation based methods, the latter conclusion due to the practical limitations on computation time. Hence we recommend the manual QTL curation come before further analysis. The establishment of genome-wide significance levels for QTLs has been addressed by much theoretical work and we recommend this theory, or a permutation analysis (such as provided by the R/QTL package), be employed before running NEO and comparing the possible causal model explanations.

References

Simulation model	proportion causal θ_{causal}	edge score	LEO.NB score Mean (SE)	threshold	Power at RT (SE)	False positive rate at RT (SE)
1a.1b.0	0.0	LEO.NB.OCA	-4.75 (0.12)	0.3		0.0041 (0.00078)
1a.1b.50	0.5	LEO.NB.OCA	-0.51 (0.09)	0.3	0.29 (0.020)	
1a.1b.100	1.0	LEO.NB.OCA	2.03 (0.08)	0.3	0.90 (0.0099)	
1a.1b.100.4an.4bn	1.0	LEO.NB.OCA	2.11 (0.07)	0.3	0.94 (0.0069)	
1a.1b.0	0.0	LEO.NB.CPA	-3.09 (0.11)	0.3		0.027 (0.0040)
1a.1b.50	0.5	LEO.NB.CPA	-0.19 (0.1)	0.3	0.38 (0.023)	
1a.1b.100	1.0	LEO.NB.CPA	1.48 (0.07)	0.3	0.83 (0.015)	
1a.1b.100.4an.4bn	1.0	LEO.NB.CPA	1.50 (0.06)	0.3	0.86 (0.013)	

Table 1: **Statistical power and false positive rates for different single edge simulation models (Figure 1a).** We report the false positive rate and power for the LEO.NB.OCA and LEO.NB.CPA scores at the recommended threshold of 0.3. We considered 4 different causal scenarios for the edge $B \rightarrow A$. Simulation model '1a.1b.0' involved one signal SNP into A, one signal SNP into B and a completely confounded A-B correlation. This completely confounded null model corresponds to having independent SNPs associated with both A and B nodes. Simulation model '1a.1b.100' used an A-B association that was 100% causal. Simulation '1a.1b.100.4an.4bn' used an A-B association that was 100% causal, but it involved four neighboring noise SNPs into A and four into B. Neighboring SNPs had correlation with the primary signal SNPs sampled from $[0.4, 0.7]$. In each simulation 100 background noise SNPs were included. Results from 300 simulated data sets are shown, with sample size N = 200 in each simulation. Restricted heritabilities (Eq. 4) were set to $h'_A = h'_B = 0.4$ and the observed correlation $cor(A, B)$ was set to 0.3. The 5% false positive threshold is the mean score (under the null hypothesis of completely confounded association; $\gamma^2 = 0.3$) plus 1.644 standard deviations. The recommended threshold of 0.3 for LEO.NB.OCA is substantially more conservative than the 5% false positive rate. At a threshold of 0.3, we obtain a false positive rate of 0.027 for the CPA score which is much higher than that of the LEO.NB.OCA method (0.0041). As aside, we mention that we recommend a threshold of 0.8 for the CPA score. The power of the CPA score under the fully causal model, while at an acceptable 83-86%, is slightly lower than that of the LEO.NB.OCA score: 90-94% for the same data. To compare the LEO.NB.CPA and the LEO.NB.OCA at the same false positive rate, a LEO.NB.CPA threshold of 1.552 would be needed to obtain the false positive rate of 0.0041 (± 0.00078 SE). At this threshold LEO.NB.CPA would yield only 48% (± 2.3% SE) power on the 100% causal simulations.

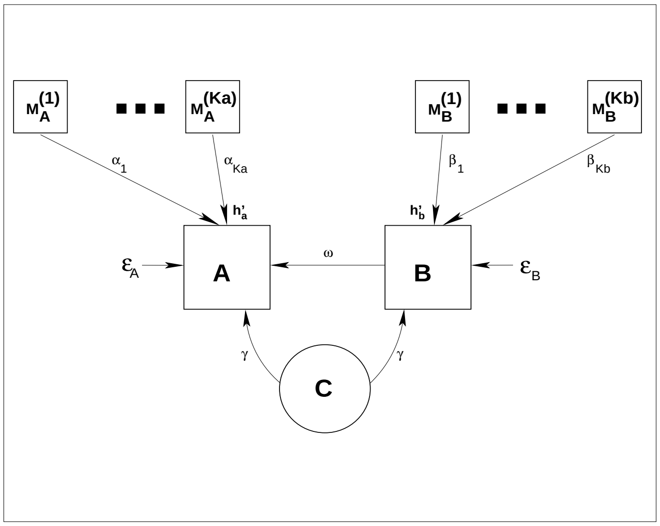

(a)

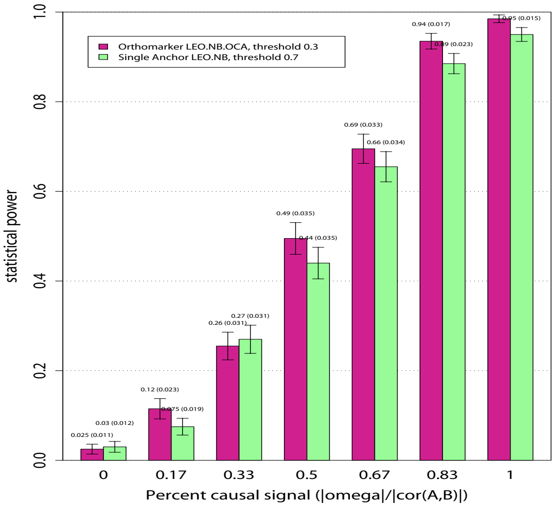

(b)

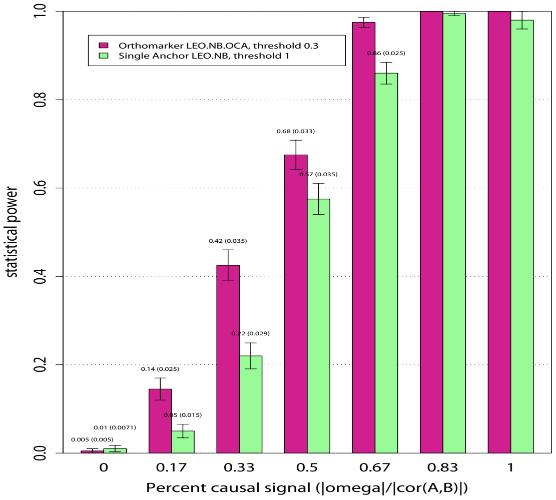

(c)

Figure 1: **Single edge simulation studies involving the LEO.NB.CPA score (Eq. 2) and the LEO.NB.OCA score (Eq. 3).** (a) outlines the parameters used in the single edge $A \leftarrow B$ simulation model. A hidden confounder C affects the correlation between A and B. The strength of confounding is determined by the path coefficient γ. The path coefficient ω parameterizes the true causal effect of B on A. The proportion of causal signal is defined as $\theta_{causal} = |\omega/cor(A, B)|$ (Eq. 6). The effect of SNP markers on traits A and B is parameterized with the restricted heritabilities h'_A and h'_B, respectively (Eq. 4). The variance in A and B due to noise is denoted by ϵ_A and ϵ_B. Figures (b) and (c) report the power of the LEO.NB.OCA score (pink bars) and the LEO.NB.CPA score (green bars) to retrieve the causal signal $A \leftarrow B$. We used a single causal anchor $M_A^{(1)}$ and $M_B^{(1)}$ for traits A and B respectively. In this case, the LEO.NB.CPA score (Eq. 2) reduces to the single marker LEO.NB score (Eq. 1). The models of Figures (b) and (c) have restricted heritabilities $h'_A = h'_B = 0.2$ and $h'_A = h'_B = 0.6$, respectively. The simulations used a fixed sample size $N = 200$ and correlation $cor(A, B) = 0.4$. When there is no causal signal ($\theta_{causal} = 0$, left most bars), the statistical power equals the false positive rate. In this case, the LEO.NB.OCA threshold of 0.3 leads to a false positive rate < 0.05. To ensure a fair comparison, we chose a threshold for LEO.NB.CPA that results in a comparable false positive rate. The LEO.NB.OCA score tends to have higher power than the LEO.NB.CPA score.

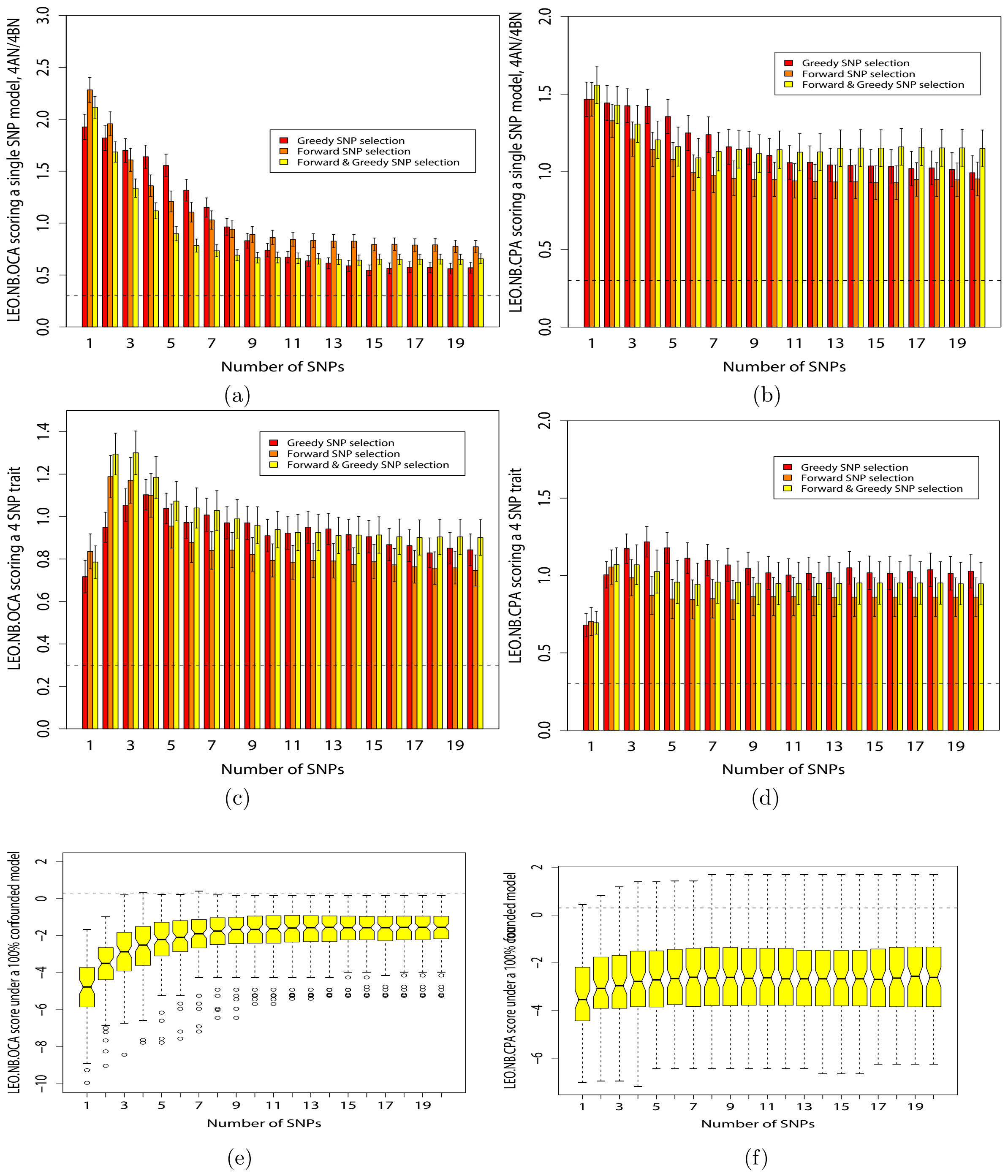

Figure 2: **Simulation study for evaluating the robustness of LEO.NB scores with respect to the addition of extraneous noise SNPs**. Figures (a-f) show the dependence of the LEO.NB scores (y-axis) on the number of added SNPs (x-axis). Figures (a,c,e, first column) and (b,d,f) correspond to the LEO.NB.OCA and LEO.NB.CPA scores, respectively. Figures (a,b, first row) and (c,d) correspond to models with 1 and 4 true signal SNPs, respectively. Note that the LEO.NB scores peak close to the true number of signal SNPs even in the presence of noise SNPs. Figures (a,c) show that the LEO.NB.OCA score is robust to the addition of extraneous noise SNPs, as the score stays above the recommended 0.3 threshold (dashed horizontal line). Figures (e,f) correspond to a model with no causal signal ($M_A \rightarrow A \leftarrow C \rightarrow B \leftarrow M_B$). The LEO.NB.OCA score remains below 0.3.
